# Optimisation of a Topical Miltefosine Carbopol-Based Gel for Cutaneous Leishmaniasis: Antileishmanial Activity After Storage and Drug Response

**DOI:** 10.3390/gels12090779

**Published:** 2026-08-31

**Authors:** Diana Paola Peña, Laura F. Neira, Patricia Escobar

**Affiliations:** Centro de Investigación de Enfermedades Tropicales (CINTROP-UIS), Departamento de Ciencias Básicas, Escuela de Medicina, Universidad Industrial de Santander, Bucaramanga 680002, Colombia; laurafernandaneira@hotmail.com

**Keywords:** cutaneous leishmaniasis, miltefosine, topical formulation, carbopol-based gel, pharmaceutical stability, dose–response, drug development

## Abstract

We previously demonstrated the therapeutic potential of a 0.5% miltefosine carbopol-based gel (G-MTF) in experimental cutaneous leishmaniasis (CL). To support formulation optimisation before clinical evaluation, this study assessed whether G-MTF retained antileishmanial activity after storage at different temperatures and durations and evaluated its dose–response following topical application to CL lesions in BALB/c mice infected with *Leishmania amazonensis*. Storage effects on some physicochemical and morphological properties, sterility, and MTF detection were also assessed. In vitro antileishmanial activity was largely maintained, although prolonged storage at 35–45 °C moderately reduced potency. During 60 days of follow-up, CL lesions reactivated in 17% of the 118 treated mice, with most reactivations occurring after treatment with G-MTF formulations stored for 3–6 months at 35–45 °C. Dose–response analysis showed concentration-dependent effects on lesion reduction and parasite burden, with estimated ED_50_ values of 3.64 and 4.02 mg/kg, respectively. All carbopol-based gel formulations remained sterile and retained physicochemical characteristics at 8–28 °C, with pH values within the normal skin range. Storage at 8 °C increased viscosity and reduced spreadability, whereas higher temperatures increased swelling. MALDI-TOF MS demonstrated that MTF remained detectable after prolonged storage, while SEM showed preserved morphology at 8–28 °C. Overall, carbopol-based G-MTF maintained therapeutic activity and acceptable physicochemical characteristics for up to six months at 8–28 °C. Validated quantitative stability-indicating and drug-release methods are needed to confirm MTF chemical stability and drug availability in the carbopol-based gel.

## 1. Introduction

Cutaneous leishmaniasis (CL) is a neglected vector-borne disease caused by protozoa of the genus *Leishmania* and transmitted by infected female phlebotomine sandflies. More than one billion people live in endemic areas worldwide, with over one million new cases reported annually. Although CL rarely leads to death, it causes ulcerative skin lesions that can result in permanent scarring, psychological distress, and social stigma, particularly among vulnerable populations. About 95% of cases occur in the Americas, the Mediterranean basin, the Middle East, and Central Asia. In the Americas, the causative species include *Leishmania (V.) braziliensis*, *Leishmania (V.) panamensis*, *Leishmania* (L.) *mexicana*, *Leishmania (V.) guyanensis* and *Leishmania* (L.) *amazonensis*. Treatment choice depends on the disease form, parasite species, geographic location, patient factors (such as age, pregnancy, and immune status), and drug-related issues (such as dose, stability, and toxicity). Current options include pentavalent antimonials (Sb^V^) given intralesionally, intramuscularly, or intravenously; oral miltefosine; pentamidine isethionate; local treatments such as thermotherapy or topical paromomycin; and liposomal amphotericin B administered intravenously [1]. Systemic therapies are often limited by toxicity, prolonged treatment regimens, high costs, emerging resistance, and restricted accessibility in endemic regions. Topical formulations have emerged as a promising alternative, offering localised drug delivery, reduced systemic exposure, improved safety, and enhanced patient adherence [2].

Miltefosine (MTF) is a synthetic alkylphosphocholine and the first orally active agent approved for the treatment of leishmaniasis. For CL, the usual oral dose is 2.5 mg/kg daily for 28 days; however, reported cure rates vary widely (approximately 33% to 91%), reflecting marked heterogeneity across *Leishmania* species, host immune status, pharmacokinetic variability and geographical setting [3,4,5,6]. Additionally, a higher rate of treatment failure has been observed in pediatric patients who mostly displayed higher MTF concentrations in their bodies than in adults receiving the same weight-based dose [7]. Beyond its direct antiparasitic activity, MTF exerts immunomodulatory effects, including enhancing macrophage activation and modulating pro-inflammatory cytokine responses. Miltefosine pharmaceutical capsules are generally stable under recommended storage conditions, although the compound is hygroscopic and moisture-sensitive [8]. Miltefosine is characterised by high chemical and metabolic stability and undergoes limited biotransformation in vivo, potentially via phospholipase-mediated pathways [9]. Consequently, the drug is eliminated slowly, with an initial half-life of about 7 days and a terminal half-life close to 30 days [9,10]. This prolonged persistence leads to gradual tissue accumulation during the standard 28-day treatment course [9]. Mild to moderate side effects such as gastrointestinal distress (vomiting, nausea and diarrhoea), transient elevations in transaminases and creatinine levels, and teratogenic effects in experimental models have been reported after oral MTF administration [11,12].

Our earlier research showed that a Carbopol^®^-based topical gel containing miltefosine (G-MTF) effectively treats experimental CL caused by various New World *Leishmania* species [13]. In BALB/c mice infected with *L. amazonensis*, topical G-MTF promoted complete healing of lesions, prevented relapse, induced re-epithelialisation and hair regrowth, reduced IL-4, TNF-α, and VEGF production, and modulated the local inflammatory response, with no evidence of significant local toxicity [14]. These results established G-MTF as a promising candidate for CL in humans. More broadly, carbopol-based gel-drug delivery systems can prolong drug residence at the application site, enable localised delivery, and potentially reduce systemic exposure [15]. Polysaccharide and other biopolymer-based hydrogels are particularly promising because of their biocompatibility, tunable physicochemical properties, and ability to enhance drug retention. Recent studies have developed multifunctional hydrogels with controlled drug release, antimicrobial, antioxidant, and tissue-regenerative properties [16,17].

Nevertheless, available studies on topical MTF have primarily focused on formulation development, biological activity, and, in some cases, skin permeation, while evidence regarding the preservation of therapeutic activity during storage remains limited [18,19]. In vitro studies have generally reported limited MTF skin permeation, although the extent of permeation varies according to the skin or membrane model, formulation, and experimental conditions. For example, low overall permeation was reported in mouse skin in Franz diffusion studies, whereas ultradeformable liposomes increased MTF permeation through human skin compared with free MTF [20,21,22]. To our knowledge, MTF permeation from a carbopol-based gel has not been characterised. Studies evaluating the preservation of therapeutic activity of topical formulations during storage remain scarce, particularly in leishmaniasis. Such data are important for formulation optimisation, shelf-life determination, and eventual clinical translation. Changes in the formulation matrix or drug properties during storage may affect formulation performance and therapeutic efficacy, highlighting the need for detailed pharmaceutical characterisation [2,15,16,17,18,19,23].

Dose–response studies are also important for optimising topical formulations and identifying MTF concentrations associated with therapeutic activity. In the present study, we evaluated the concentration–response relationship of G-MTF based on lesion reduction and parasite burden following topical treatment in an experimental model of CL. This is particularly relevant because CL lesions differ substantially from normal skin. Histopathologically, CL lesions show epidermal and dermal alterations, including acanthosis, spongiosis, hyperkeratosis, parakeratosis, and ulceration, along with inflammatory cell infiltration by lymphocytes, plasma cells, and macrophages, some of which contain intracellular parasites [14]. These infection-associated changes may influence drug penetration and tissue retention compared with healthy skin [24]. Thus, defining the concentration–response relationship in the infected lesion model provides a useful basis for further optimisation of topical MTF formulations.

The aim of this study was to evaluate whether G-MTF retained antileishmanial activity after storage at different temperatures and durations and to assess its dose–response relationship following topical application to CL lesions in BALB/c mice infected with *L. amazonensis*. The effects of storage on selected physicochemical and morphological properties, sterility, and MTF detection were also assessed. This study was designed to provide complementary in vitro and in vivo evidence of the biological performance of G-MTF during storage and to support further optimisation of the formulation for topical treatment of CL. Skin permeation was not an objective of the present study, and a reliable quantitative method for determining MTF in receptor samples or in the carbopol-based gel formulation could not be established. A definitive assessment of MTF chemical stability during storage will require a validated quantitative stability-indicating method.

## 2. Results and Discussion

### 2.1. Physicochemical Evaluation of G-MTF After Different Incubation Times and Temperatures

**Figure 1:**
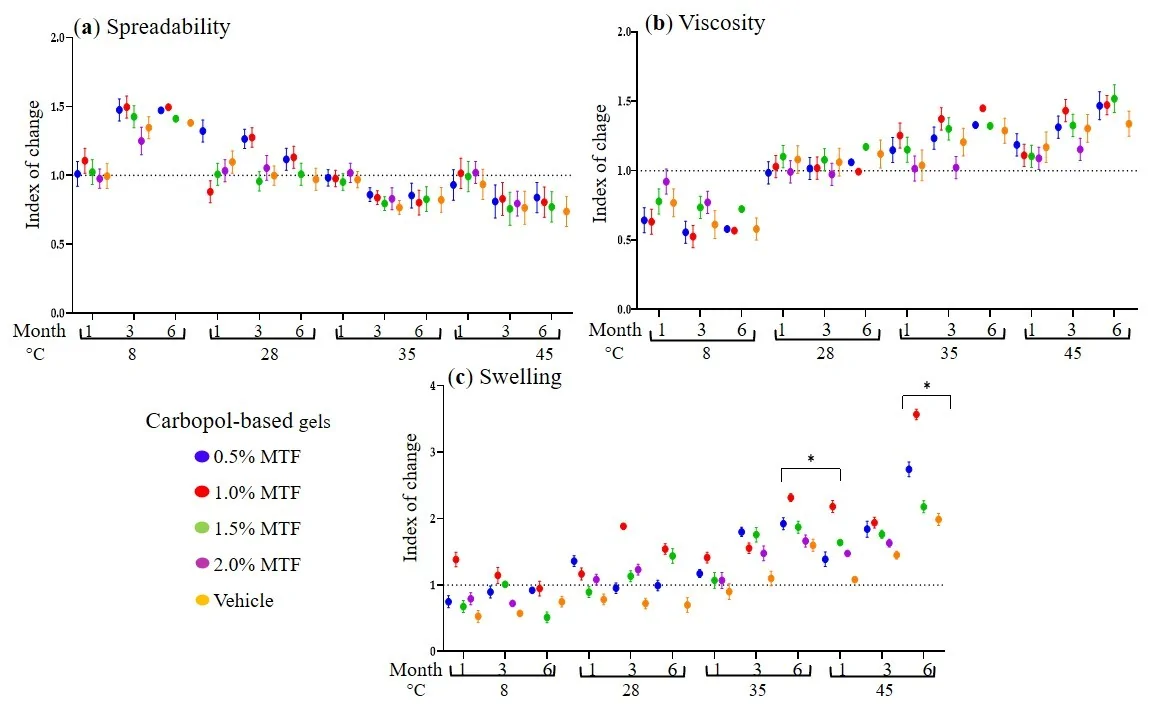
Effect of storage conditions on the physicochemical properties of G-MTF carbopol-based gel. Changes in spreadability (**a**), viscosity (**b**), and swelling (**c**) of 0.5–2.0% G-MTF stored at 8, 28, 35, and 45 °C for 1, 3, and 6 months. Results are expressed as an Index of Change relative to freshly prepared formulations (value at time x/fresh value); values <1 and >1 indicate decreases and increases, respectively. Data are presented as mean ± standard deviation (SD) (*n* = 3). Statistical analysis was performed using one-way ANOVA. Statistical significance was considered at * = *p* < 0.05. Raw data are provided in Tables S1–S5.

In this work, Carbopol^®^ Ultrez 10 NF was selected as the gelling agent because it is a pharmaceutical-grade carbomer widely used in topical formulations for human use. In addition to its established safety profile (it is synthesised in a toxicologically safe, benzene-free system), it provides effective thickening, good bioadhesion, and rheological properties [25,26]. Overall, the physicochemical properties of the G-MTF formulations were retained during storage, particularly at 28 °C, supporting their potential use in endemic regions where cold-chain storage may be limited [27]. The carbopol-based gels retained their translucent, semi-solid form. However, after prolonged storage, they exhibited slight solidification at 8 °C and partial liquefaction at 45 °C (Table S1). These observations align with the temperature-dependent rheological behaviour of carbopol-based gels, in which changes in polymer chain mobility, hydration, and molecular interactions can affect their structural organisation and viscosity [26,28]. The pH values of G-MTFs were similar (Table S2). Given that the skin surface is naturally acidic, with an average pH of approximately 4.7 [29], the prepared gels were considered suitable for topical application, thereby maintaining skin barrier integrity and minimising the risk of irritation and other local adverse effects [30,31]. The effects of spreadability (SP), viscosity (V), and swelling (SW) are shown in Figure 1. For compression, an Index of Change was calculated by dividing the sample test value by the value of the just-prepared G-MTF; values less than 1 indicate a decrease, and values greater than 1 indicate an increase in the variable relative to the just-prepared G-MTF. (Figure 1). At 8 °C, SP decreased, and V increased (Table S4). At the higher temperatures, the opposite was observed (Tables S3 and S4). At 28 °C, variability was lower for both SP and V, with slight increases. This behaviour is consistent with the established rheological properties of carbomer-based hydrogels, in which increased temperature enhances polymer chain mobility and reduces resistance to flow by altering hydration and intermolecular interactions within the network [26,31]. Structurally, this response is governed by thermally induced expansion of free volume and accelerated relaxation of the microgel network [28,30,32]. Notably, only minor fluctuations were observed at 28 °C. This is consistent with reports indicating that carbomer-based dispersions maintain a consistent polymer network architecture and exhibit limited viscosity variation at typical room temperature [26,30,32]. Regarding swelling, the 1.0% formulation showed an increase after 3 months (Tables S3–S5). Swelling behaviour increased at elevated temperatures after prolonged storage. This response reflects temperature-dependent changes in the carbopol-based gel network associated with increased water uptake and polymer relaxation. The gels dissolved under acidic conditions and precipitated under basic conditions but remained intact in peroxide solution (Table S6). Mass loss was generally comparable among formulations but was greater at 8 and 45 °C (Table S7). Although changes in swelling and rheological properties may potentially affect drug diffusion and release, these effects were not directly evaluated in the present study. Future studies incorporating drug-release and skin-permeation assays will be needed to determine their influence on MTF delivery. In addition, longer-term studies under ICH-recommended conditions and using validated stability-indicating methods will be needed to establish the shelf-life and specifications for future development [33].

### 2.2. Morphological Analysis of Carbopol-Based Gels by Scanning Electron Microscopy (SEM)

SEM analysis showed that fresh and stored 0.5% MTF carbopol-based gels and the vehicle exhibited a relatively homogeneous, porous structure with interconnected filamentous networks, consistent with a well-developed polymeric matrix (Figure S1). Formulations stored at 8 °C and 28 °C retained a morphology similar to that of the freshly prepared carbopol-based gel, with filament thicknesses ranging from 29.14 to 99.8 nm, indicating good structural stability. In contrast, storage at 35 °C resulted in greater condensation of the polymeric network, possibly related to water loss and the physicochemical changes observed during storage. The vehicle exhibited a morphology similar to that of the MTF-containing carbopol-based gel, suggesting that the observed structure was primarily associated with the carbopol matrix. Although the morphology of carbopol-based gels may influence swelling and drug release [34], these relationships were not directly evaluated in the present study.

### 2.3. Sterility Test of the G-MTF

All G-MTF formulations remained free of microbial contamination throughout the 6-month evaluation period. The absence of microbial growth throughout the study suggests that appropriate preparation and handling conditions were maintained. In addition, the use of DMSO (10% as a permeant) and, especially, sodium benzoate (0.1% [13]) may have helped preserve microbiological stability during storage, consistent with its established use as an antimicrobial preservative in topical formulations [32]. This is consistent with the requirements for topical preparations in pharmacopeial texts and suggests that the combined effect of preservatives, DMSO, and reduced water activity effectively prevents microbial growth [35].

### 2.4. Mass Spectrometry Detection of MTF During Storage

**Figure A1:**
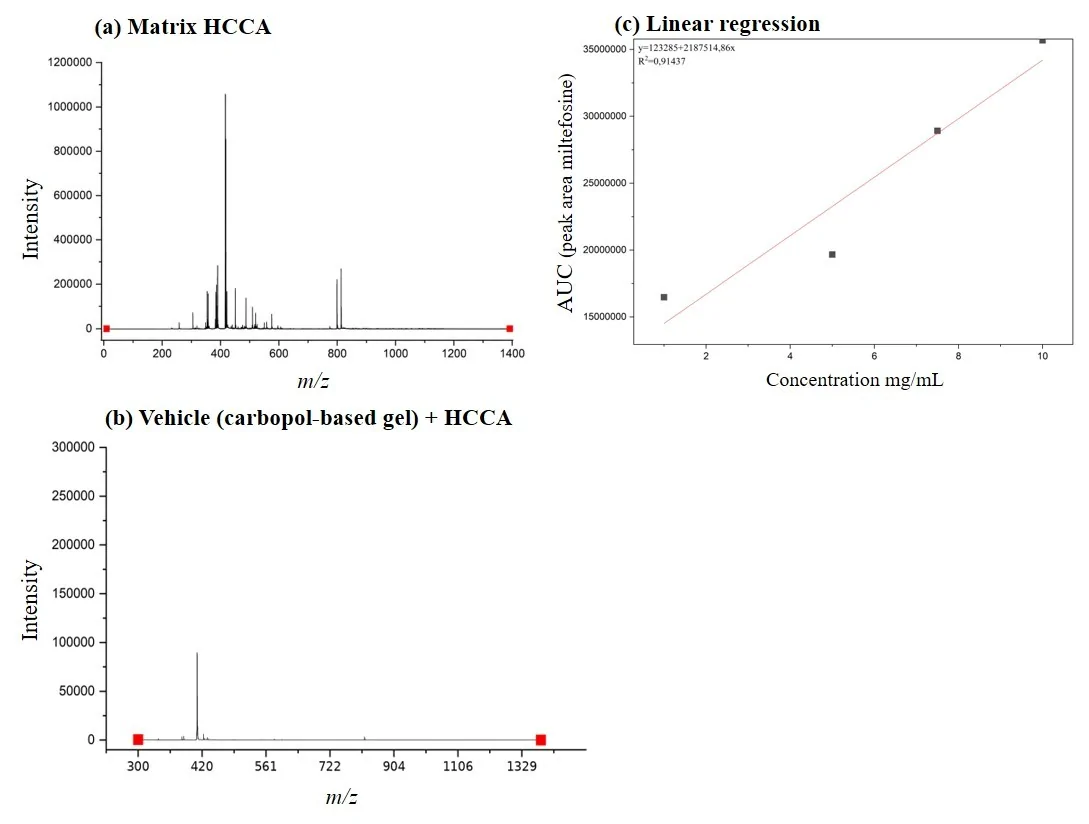
MALDI-TOF MS method validation for miltefosine analysis. (**a**) Representative MALDI-TOF mass spectrum of the α-cyano-4-hydroxycinnamic acid (HCCA) matrix. (**b**) Representative MALDI-TOF mass spectrum of vehicle or blank carbopol-based gel. (**c**) Calibration curve generated using pharmaceutical-grade miltefosine standards (1–10 mg/mL), yielding the regression equation y = 123,285 + 2,187,514.86x (R^2^ = 0.91437).

**Figure A2:**
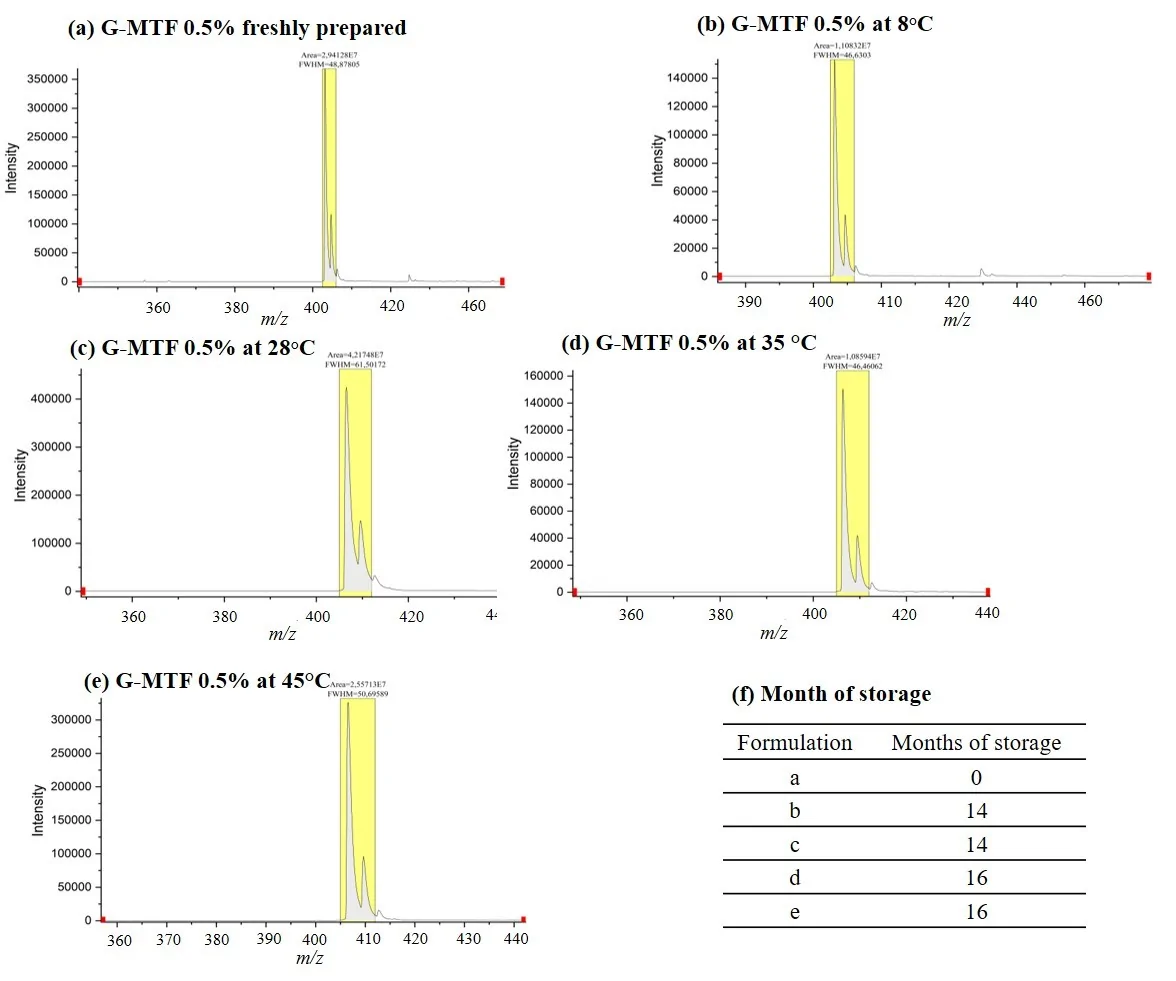
Representative MALDI-TOF spectra of freshly prepared G-MTF 0.5% at 8, 28, 35, and 45 °C for different periods of time. The integrated peak area is shown and highlighted in yellow. The table shows the storage period (month) for each 0.5% G-MTF.

**Figure A3:**
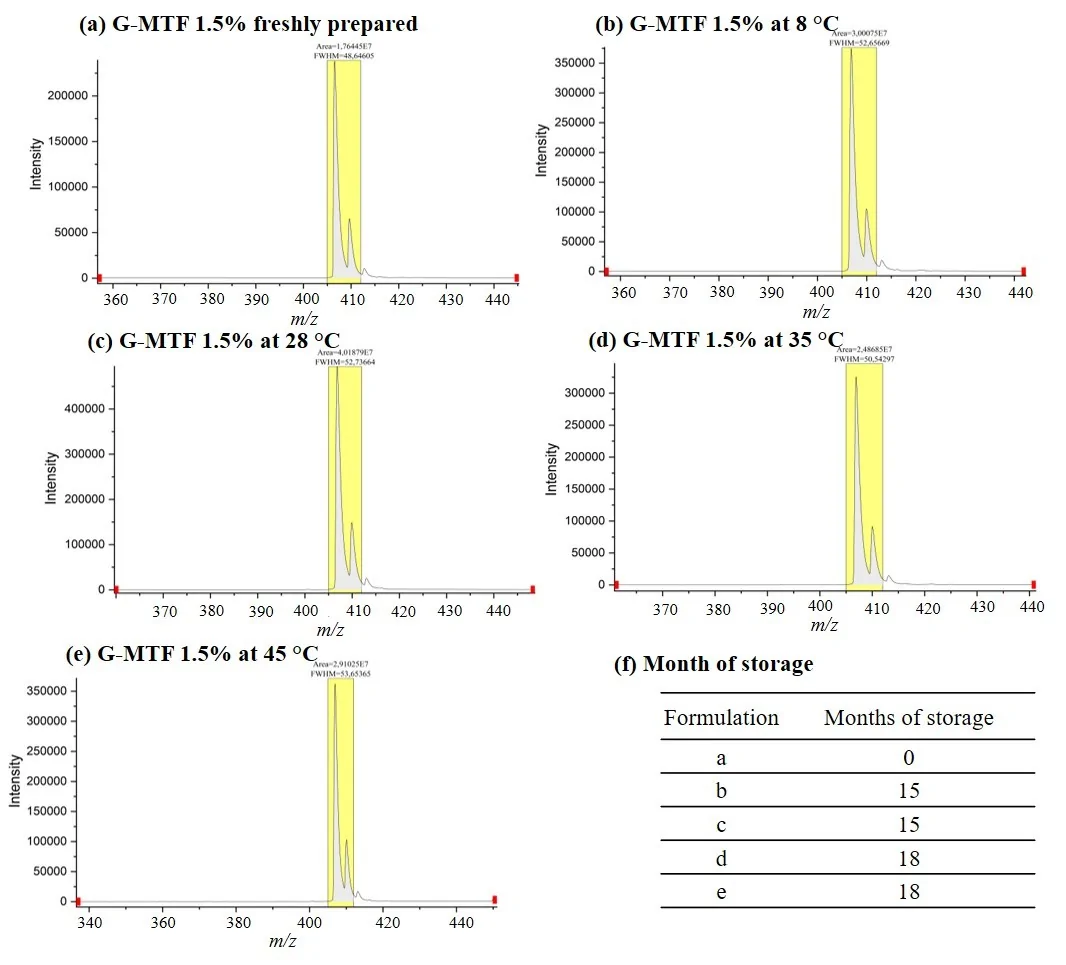
Representative MALDI-TOF spectra of freshly prepared G-MTF 1.5% at 8, 28, 35, and 45 °C for different periods of time. The integrated peak area is shown and highlighted in yellow. The table shows the storage period (month) for each 1.5% G-MTF.

MTF was detected by MALDI-TOF MS following co-crystallisation with the HCCA matrix. The HCCA matrix alone and with the vehicle (blank carbopol-based gel) showed characteristic low-molecular-weight ions (Figure A1a,b), whereas MTF produced a distinct protonated ion at *m*/*z* 408.3, enabling selective detection in the 0.5% G-MTF (Figure A2a–e) and 1.5% G-MTF (Figure A3a–e) formulations. A semi-quantitative calibration curve yielded the regression equation y = 123,285 + 2,187,514.86 × (R^2^ = 0.91437; Figure A1c), which raises concerns about its use to calculate MTF concentration. Under the conditions of this study, MALDI-TOF MS was unable to accurately quantify MTF stability in gel samples during storage. Nonetheless, it proved useful for detecting MTF levels in the gels over time. The absence of an MTF signal in drug-free controls (vehicle) verified that the detected signals were from MTF itself, not the gel matrix. While MTF signals can be detected and compared between samples, factors such as ionisation efficiency, sample preparation, and matrix crystallisation can influence signal intensity. This limits the ability to directly relate signal strength to MTF concentration [36,37]. Consequently, fluctuations in signal intensity during storage do not necessarily indicate proportional changes in MTF content. Accurate quantitative analysis would require a validated method with calibration and internal standards [38]. Overall, MALDI-TOF MS was effective for detecting MTF and monitoring its spectral profile over time, but it could not precisely quantify the percentage of MTF remaining in the gel. These findings highlight the need for validated analytical methods to assess the long-term stability of MTF in carbopol-based gel (e.g., HPLC-UV and LC-MS/MS) and to establish appropriate storage conditions and shelf-life specifications. Table A1 compares the MALDI-TOF MS approach used in this study with previously reported analytical methods [39,40,41].

The determination of MTF after more than one year of storage also underscores the importance of appropriate disposal of expired or unused MTF-containing carbopol-based gel. Disposal in accordance with applicable pharmaceutical waste-management procedures may help minimise the potential release of persistent drug residues into wastewater and the environment [42,43].

### 2.5. Effect of G-MTF Storage on In Vitro Antileishmanial Activity and Cell Toxicities

**Figure 2:**
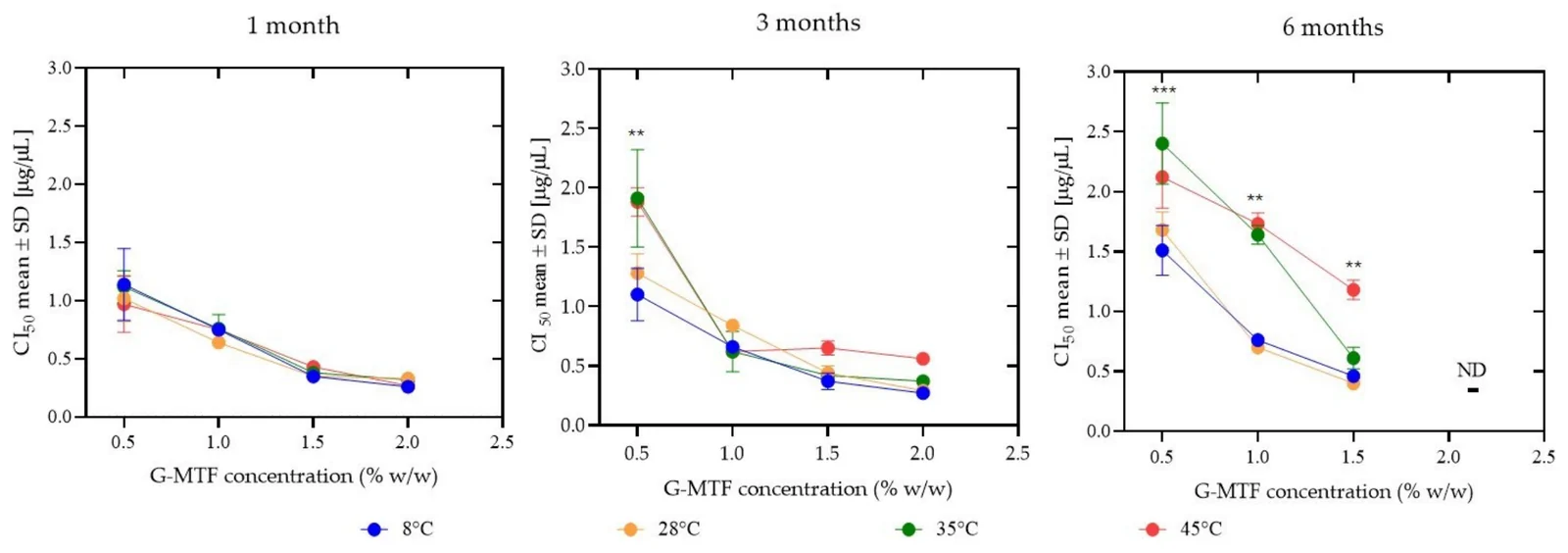
Influence of storage temperature and incubation time on the antileishmanial activity of G-MTF formulations against *L. amazonensis* promastigotes. Gels were incubated for 1, 3, and 6 months at 8, 28, 35, and 45 °C. Lower IC_50_ values indicate greater antileishmanial activity. Three independent experimental assays were performed. Statistical analysis was conducted using two-way ANOVA followed by Tukey’s multiple-comparisons post hoc test. Statistical significance was considered at ** = *p* < 0.01, and *** = *p* < 0.001, ND: not determined.

In vitro treatment with G-MTF formulations demonstrated concentration-dependent antileishmanial activity that was largely retained during storage, although prolonged exposure to elevated temperatures moderately reduced potency (Figure 2). Freshly prepared formulations exhibited a concentration-dependent response against *L. amazonensis* promastigotes, with IC_50_ values decreasing from 1.48 ± 0.12 µg/µL for the 0.5% G-MTF formulation to 0.28 ± 0.05 µg/µL for the 2.0% formulation (Table S8). Although antileishmanial activity declined slightly (1.3- to 2.7-fold) after prolonged storage, the 2.0% formulations consistently exhibited the highest potency across all storage conditions. Statistically significant changes (*p* < 0.05) were observed during storage times: for the 0.5% formulation after 3 months and for all formulations after 6 months. Storage temperature had little effect on activity after 1 month; however, prolonged storage at 35 °C and 45 °C resulted in increased IC_50_ values, indicating reduced antileishmanial potency (Figure 2). The effect of temperature on G-MTF activity is also illustrated in Figure S2. A similar concentration-dependent pattern was observed against intracellular amastigotes, with lower IC_50_ values at higher concentrations (1.58 ± 0.20 µg/µL at 1.5% versus 2.06 ± 0.10 µg/µL at 0.5%). Storage at elevated temperatures led to a moderate increase in IC_50_ values, particularly at 45 °C (Table S9). The observed activity of G-MTF against *Leishmania* was consistent with the intrinsic antileishmanial properties of free MTF and its well-documented dose-dependent efficacy against *Leishmania* spp. in vitro, whereby increasing drug concentrations result in greater parasite susceptibility and correspondingly lower IC_50_ values [44,45,46]. Accordingly, the superior potency of the 1.5% formulation likely reflects the higher MTF concentration in the system. Despite reduced activity under accelerated storage conditions (45 °C), the formulations retained measurable antileishmanial activity. The greater variability observed for the 0.5% formulation may be related to its lower drug concentration, which can increase sensitivity to experimental variability and minor storage-related changes, thereby affecting the consistency of the biological response over time.

Cytotoxicity of the G-MTF in THP-1 cells increased with MTF concentration, with CC_50_ values of 1.88 ± 0.21, 0.92 ± 0.04, and 0.82 ± 0.14 µg/µL for 0.5%, 1.0%, and 1.5%, respectively. A slight increase in CC_50_ after storage was observed, particularly for the 0.5% formulation, rising from 1.88 ± 0.21 µg/µL (freshly prepared) to 2.62 ± 0.25 µg/µL after 6 months at 45 °C, indicating reduced cytotoxicity over time (Table S10). The selectivity index (SI) was higher for the 1.5% formulation (≥2.0 under most conditions), reaching 3.03 at 35 °C (1 month), whereas lower concentrations frequently showed SI < 2.0 (Table S11). In vitro results showed SI values below 3, indicating limited selectivity toward *Leishmania* relative to THP-1 cells. The vehicle (gel without MTF) exhibited cytotoxicity, with a CC_50_ of 9–13 µg/µL, indicating that the formulation itself contributes to the observed cytotoxic response. However, the vehicle showed no antileishmanial activity at the highest concentration tested (IC_50_ > 16 µg/µL). Therefore, the SI values calculated for the MTF-loaded carbopol-based gel reflect the biological activity of the complete formulation and cannot be attributed solely to MTF. Importantly, topical application of the formulations to healthy BALB/c mice produced no visible signs of irritation or corrosion in vivo [13].

### 2.6. Effect of G-MTF Storage on In Vivo Antileishmanial Activity

**Figure 3:**
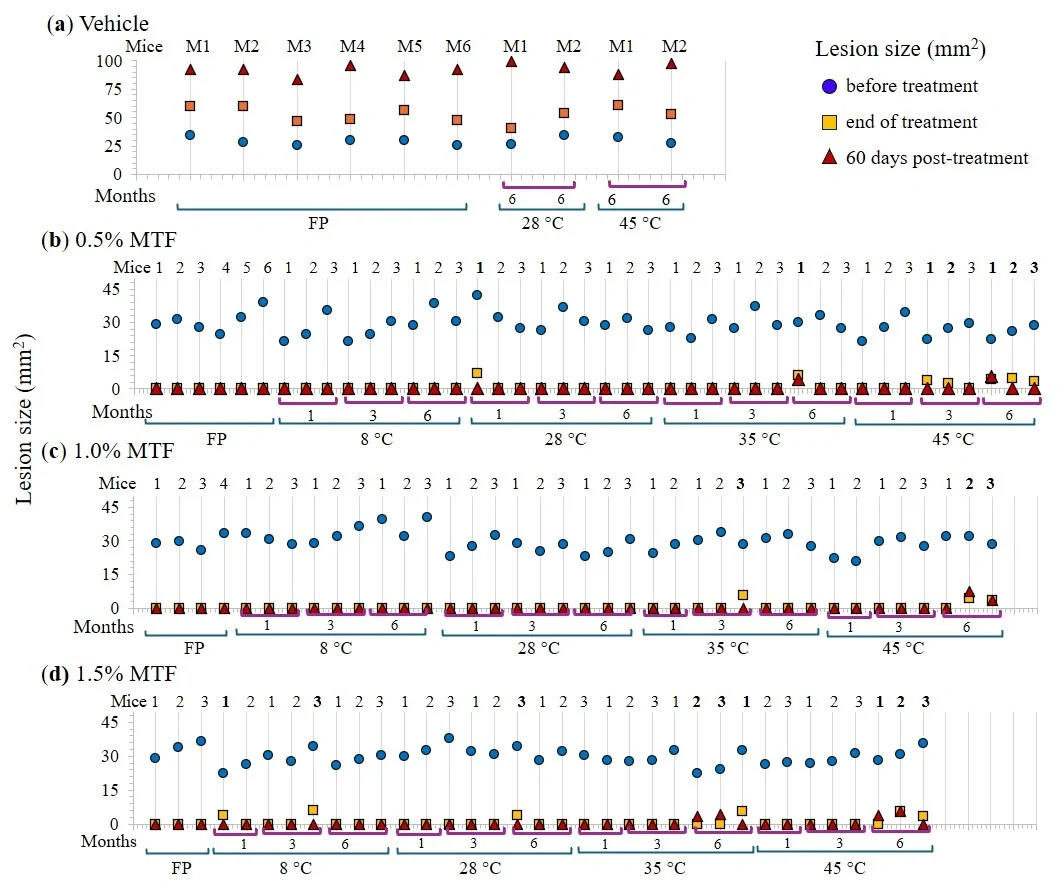
Effect of G-MTF formulations on *L. amazonensis*-infected mice after storage under different conditions. The figure displays the results for each individual mouse. Lesion size (LS, mm^2^) in each individual mice, measured before treatment (blue circles), at the end of treatment (orange square), and 60 days post-treatment (red triangle) in mice treated with hydrogen vehicle (**a**) and G-MTF at 0.5% (**b**), 1.0% (**c**), 1.5% (**d**), freshly prepared (FP) and after storage at 8, 28, 35, and 45 °C for 1, 3, and 6 months.

The effect of storage conditions on the in vivo efficacy of G-MTF formulations was assessed by measuring lesion size (LS) at baseline, at the end of treatment, and 60 days post-treatment (Figure 3). Freshly prepared formulations achieved complete lesion resolution (LS = 0) at all tested concentrations, with no relapse during follow-up (Figure 3). Similar efficacy was maintained after storage at 8 °C and 28 °C, where most formulations produced complete lesion healing and sustained cure (Figure 3). In contrast, prolonged storage at elevated temperatures progressively reduced therapeutic efficacy. At 35 °C, residual lesions were observed after 6 months of storage, particularly in the 0.5%, 1.0%, and 1.5% formulations, and relapses were detected in the 1.5% group during follow-up. The greatest loss of efficacy occurred in G-MTF stored at 45 °C, where lesion resolution remained complete after 1 month of storage, but residual lesions and relapse became evident after 6 months, indicating a time- and temperature-dependent decline in therapeutic performance. Control groups (0% MTF) showed progressive disease, with lesion sizes increasing throughout the study, confirming the absence of spontaneous healing.

Overall, complete lesion resolution was maintained in most groups treated with freshly prepared formulations and in those stored at 8 °C or 28 °C, whereas prolonged storage at 35 °C and 45 °C was associated with residual lesions and occasional relapse, indicating reduced in vivo efficacy under accelerated storage conditions. To our knowledge, few studies have investigated the impact of long-term storage conditions on the in vivo efficacy of topical antileishmanial formulations in experimental CL. While previous studies have demonstrated the therapeutic efficacy of topical miltefosine formulations in experimental models of CL, including carbopol-based gels, liposomal systems, and other topical delivery platforms, in infected BALB/c mice [13,14,20,47]. The influence of prolonged storage at different temperatures on subsequent in vivo efficacy has remained largely unexplored. Therefore, the present findings provide evidence that G-MTF formulations can retain substantial therapeutic activity after prolonged storage, particularly at 8 °C and 28 °C, supporting their potential applicability in endemic settings with variable storage conditions.

### 2.7. Dose–Response Experiments: Lesion Size Reduction and Parasite Loads

**Figure 4:**
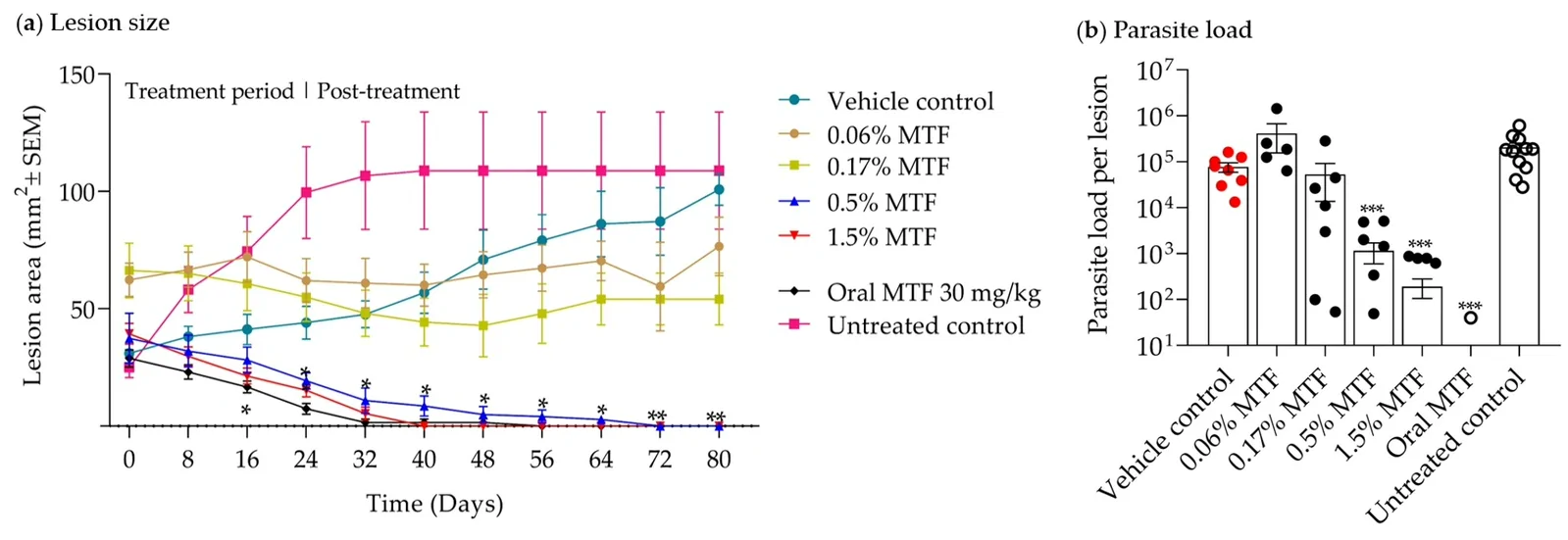
Therapeutic effects of topical G-MTF in BALB/c mice infected with *L. amazonensis*. Therapeutic efficacy of G-MTF at 0.06%, 0.17%, 0.5%, or 1.5%, oral MTF (30 mg/kg), vehicle, or untreated controls was evaluated by monitoring lesion size (mm^2^ ± standard error of the mean, SEM) over the treatment period (**a**), and parasite load per lesion measured by qPCR at the end of follow-up period. Red, black, and white points represent individual mice in the respective experimental groups. (**b**). Data were expressed as mean ± SEM. Statistical significance was assessed using one-way ANOVA with Dunnett’s post hoc test (**a**) or the Kruskal–Wallis test with Dunn’s multiple-comparisons post hoc test (**b**). * *p* < 0.05, ** *p* < 0.01, and *** *p* < 0.001 versus the vehicle control group

The study shows that a carbopol-based topical G-MTF gel effectively treats CL in mice, consistent with earlier findings on MTF activity [13,14,20,47]. After 60 days, LS dropped from 37–39 mm^2^ to 0 mm^2^ with 0.5% and 1.5% G-MTF. Oral MTF for 15 days also reduced LS. Conversely, 0.06% and 0.17% G-MTF caused LS changes from 66 to 60 mm^2^ and 77 to 102 mm^2^ during follow-up (Figure 4a). Vehicle increased LS from 31 to 101.4 mm^2^ in treated mice and from 25.07 ± 4.3 to 138.47 ± 27.43 mm^2^ in untreated mice. Parasite load at follow-up is shown in Figure 4b. It was found that 0.5% and 1.5% G-MTF significantly reduced parasite counts (*p* < 0.001) compared with the vehicle. Oral MTF also lowered parasite load (2.66 ± 2.6 parasites per lesion) with no difference from 1.5% G-MTF (*p* > 0.05). The 0.17% MTF group showed a 31.52% reduction (52,613 ± 38,822 parasites), while 0.06% MTF did not significantly reduce parasite counts (412,701 ± 257,718 parasites).

**Figure 5:**
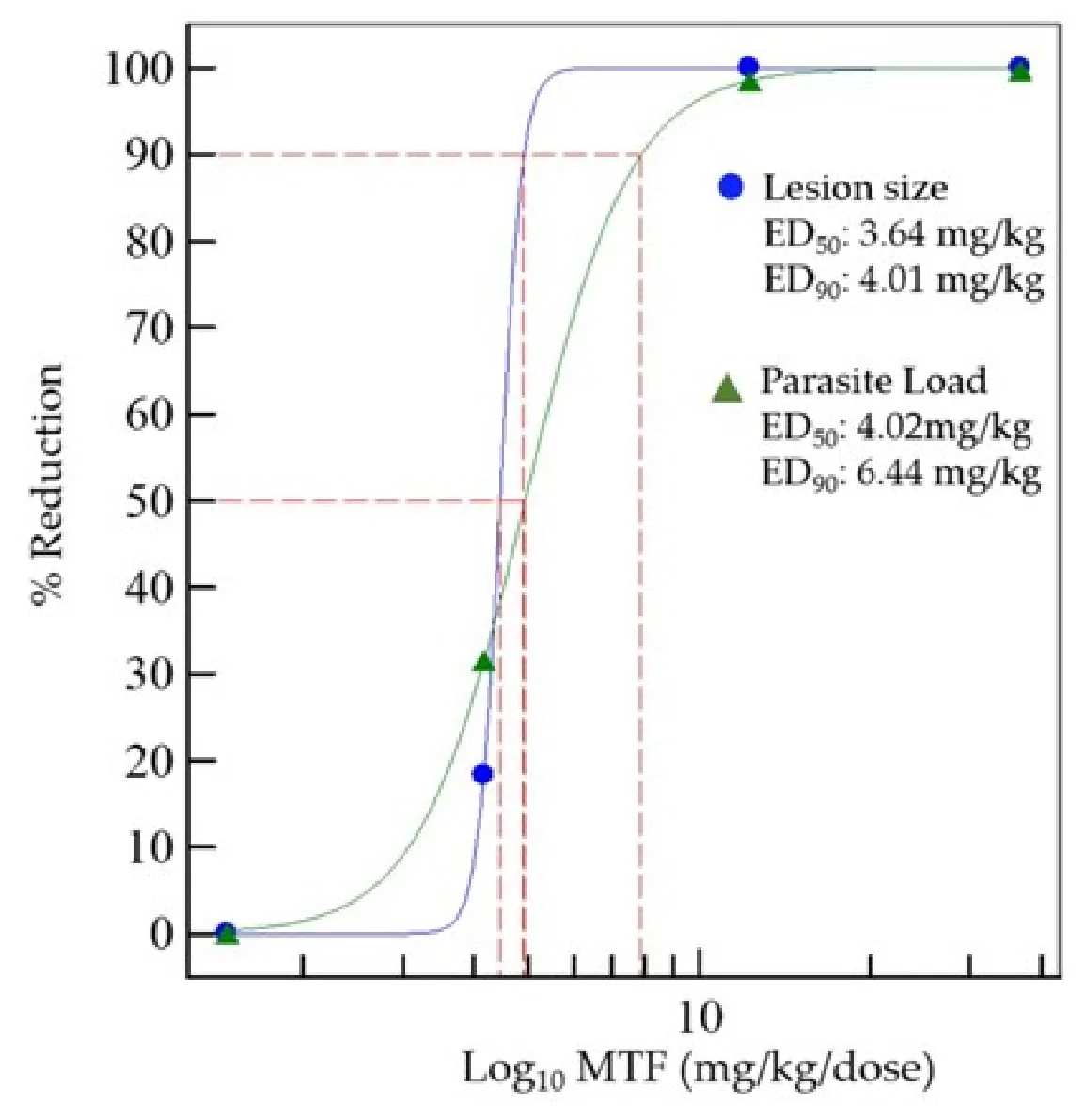
Dose–response analysis of topical G-MTF in BALB/c mice infected with *L. amazonensis*. Sigmoidal dose–response curves showing the therapeutic efficacy of G-MTF in lesion size and parasite load reduction after the follow-up period. Blue circles represent lesion size reduction, and green triangles represent parasite load reduction. Dashed red lines indicate the extrapolation used to estimate the ED_50_ and ED_90_ values for both parameters.

Topical G-MTF treatment showed a concentration-dependent effect on both lesion size (LS) and parasite burden. The ED_50_ and ED_90_ values calculated for lesion size (LS) and parasite load at the end of the experiment are shown in Figure 5. Additionally, we calculated the ED_50_ values for LS reduction during the experiment (Figure S3). For LS reduction, the ED_50_ after 25 days was 14.9 mg/kg/dose MTF (Figure S3c). Over time, the drug’s effectiveness increased, with lower MTF doses needed to reduce LS by 50% or 90% (EC_50_ from 6.32 mg/kg/dose to 3.64 mg/kg/dose MTF and ED_90_s from 15.25 mg/kg/dose to 4.01 mg/kg/dose MTF) (Figure S3d–i).

In topical drug delivery, the relationship between the applied drug concentration and therapeutic response can be influenced by the skin’s barrier properties. Therefore, increasing the applied dose does not necessarily lead to proportional increases in drug exposure at the site of infection. This is particularly relevant to topical antileishmanial therapy, in which sufficient drug exposure within infected skin is required to achieve therapeutic activity while minimising local toxicity [2,48]. In the present study, dose–response experiments demonstrated a concentration-dependent increase in the therapeutic activity of topical MTF. Sigmoidal analyses of lesion size and parasite burden confirmed a concentration–response relationship. Formulations containing 0.5% and 1.5% MTF produced marked reductions in both lesion size and parasite burden, whereas lower concentrations (0.06% and 0.17%) showed little or no therapeutic effect, suggesting a minimum concentration range associated with measurable efficacy. These findings support further optimisation of MTF concentration in the carbopol-based gel formulation to maximise therapeutic benefit while maintaining tolerability. Skin permeation and MTF deposition within the lesions were not directly evaluated in this study; therefore, the mechanisms underlying the observed concentration–response relationship cannot be confirmed. Moreover, CL lesions differ histopathologically from normal skin, with epidermal alterations and dermal inflammatory cell infiltration, including macrophages, lymphocytes, and plasma cells, as previously demonstrated in our experimental model [14]. These infection-associated changes may affect drug penetration and tissue retention compared with healthy skin and should be considered when interpreting topical drug-delivery studies [24]. Nevertheless, several properties of MTF and the formulation may have contributed to the greater therapeutic activity observed at higher concentrations. MTF is an amphiphilic alkylphosphocholine that can partition into biological membranes [10,49], and its physicochemical properties may be compatible with passive skin permeation. In addition, the formulation contained 10% DMSO, a recognised penetration enhancer that can alter stratum corneum lipid organisation and facilitate drug transport across the skin [48]. DMSO may influence skin permeability; however, its contribution to MTF penetration and the observed therapeutic response was not evaluated in the present study.

To our knowledge, no previous studies have systematically characterised the dose–response relationship of topical MTF formulations for CL. Early studies with topical paromomycin suggested concentration-dependent activity [50], while dose–response relationships for oral MTF have been demonstrated in BALB/c mice infected with *Leishmania donovani* [51]. In that study, liver parasite burden decreased in a dose-dependent manner, with reported ED_50_ and ED_90_ values of 3.98 and 27.13 mg/kg/dose, respectively. Although the ED_50_ estimated for the topical formulation in the present study (3.64 mg/kg/dose) was numerically similar to that reported for oral MTF, these values should not be interpreted as directly comparable because of differences in route of administration, dosing regimen, target tissue, and pharmacokinetic profile. Oral treatment was effective after five doses, whereas the topical regimen required 25 applications, further highlighting the importance of optimising topical dosing strategies. Such optimisation may help maximise therapeutic efficacy while minimising unnecessary drug exposure, an important consideration given the prolonged half-life and known teratogenic potential of MTF and the broader concern that prolonged or suboptimal antileishmanial exposure may contribute to the selection of less-susceptible parasites [10,46,52].

## 3. Conclusions

Overall, G-MTF demonstrated sustained antileishmanial activity and a clear concentration–response relationship in experimental CL. Formulations stored at 8–28 °C retained substantial in vitro and in vivo activity for up to six months. SEM analysis further showed that the gel retained its morphological characteristics over the 8–28 °C range. In contrast, storage at 35–45 °C was associated with greater physicochemical changes, reduced antileishmanial potency, and occasional lesion reactivation, highlighting the importance of appropriate storage conditions. Dose–response analysis identified 0.5% and 1.5% G-MTF as the most active formulations and provided ED_50_ estimates for lesion reduction and parasite burden. MALDI-TOF MS demonstrated that MTF remained detectable after prolonged storage. However, validated quantitative stability-indicating and drug-release methods are needed to confirm MTF chemical stability and drug availability in the carbopol-based gel. Together, these findings support further preclinical development of G-MTF and provide a basis for future studies of drug release, permeation, and retention in infected skin, safety, and longer-term stability prior to clinical evaluation.

## 4. Materials and Methods

### 4.1. Reagents

Miltefosine (MTF) was obtained from Gold Biotechnology^®^ (St. Louis, MO, USA) and Genyx Pharmaceuticals (Hubei ChenFang Pharm & Co., Tianmen, China) as pharmaceutical-grade, with the latter kindly donated by Genyx Pharmaceuticals. Carbopol Ultrez 10 NF polymer (Lubrizol, Wickliffe, OH, USA), dimethyl sulfoxide (DMSO), sodium benzoate, methyl 4-hydroxybenzoate, triethylamine, phorbol 12-myristate 13-acetate (PMA), and resazurin were purchased from Sigma–Aldrich (St. Louis, MO, USA). RPMI medium was supplied by Gibco (Grand Island, NY, USA), along with Schneider’s insect medium and heat-inactivated fetal calf serum (FCS) from Sigma–Aldrich. Deionised water was prepared using a Milli-Q water purification system (Milli-Q^®^, Millipore, Burlington, MA, USA).

### 4.2. Leishmania Parasites and THP-1 Cells

Promastigotes of *L. amazonensis* (MHOM/BR/73/LV78) were cultured in Schneider’s medium supplemented with 10% heat-inactivated FCS at 28 °C. Before assays, species confirmation was performed by Sanger sequencing of the IST-1 gene [53]. Human THP-1 cells (ATCC, TIB-202) were maintained in RPMI 1640 with 10% hiFBS at 37 °C in 5% CO_2_. Cells were differentiated with 130 nM PMA for 48 h, then infected with promastigotes at a 1:5 ratio for 24 h at 32 °C.

### 4.3. Mice and Ethical Considerations

Female BALB/c mice, aged 8–10 weeks, were obtained from the National Health Institute in Bogotá, Colombia. They were maintained on a 12-h light/dark cycle at 22 ± 2 °C and 55 ± 5% humidity. Food and water were available ad libitum. All animal procedures were conducted in accordance with the NIH Guide for the Care and Use of Laboratory Animals (2011), with a view to minimising suffering. The study received approval from the Ethics Committee of the Industrial University of Santander (Bucaramanga, Colombia) (Act 23 of December 2019, and Act 4110 of March 2023). Mice were monitored regularly for behavioural changes and signs of stress, weighed weekly, and humanely euthanised with ketamine/xylazine followed by cervical dislocation.

### 4.4. Mice Infection, Follow-Up and Drug Effect

Mice were infected via subcutaneous injection of 5 × 10^5^ stationary-phase *L. amazonensis* promastigotes in 100 µL PBS, pH 7.2, on the dorsal side. Lesion size was measured with a digital calliper (Tracedable^®^, VWR, Radnor, PA, USA), and the lesion area (mm^2^) was calculated [14].

Sample size was determined a priori using G*Power (version 3.1.9.7), assuming α = 0.05 and 80% power, based on effect estimates from previous studies by our group: f = 0.78 (stability studies) and 0.70 (dose–response studies). Separate calculations were performed for the formulation stability and dose–response studies, using one-way ANOVA (n = 3) and nonlinear regression approaches (n = 6), respectively. Once lesions exceeded 20 mm^2^ (approximately 50 days post-infection), animals were randomly allocated to experimental groups using a computer-generated sequence, with balanced group sizes in most groups. Outcome assessment was not blinded. No animals were excluded after randomisation, and all animals were included in the final analysis. Animals received 100 mg of carbopol-based gel applied directly to the lesion once daily for 25 days (dose–response experiment) or 28 days (stability experiments), based on our previous preclinical studies.

Some groups received oral treatment with 30 mg/kg MTF via a size 22 feeding cannula for 15 days, while others received the vehicle (without MTF) or remained untreated [14,51]. The mice were monitored for behavioural changes and signs of stress, and their weights were recorded. Lesion size (LS) was measured weekly for up to 60 days post-treatment. Results were expressed as mean ± SEM of lesion area (mm^2^) and documented photographically. Parasite load was measured by qPCR using the method described by Neira et al. (2023) [14]. The procedure involved an initial denaturation at 50 °C for 2 min, followed by 2 min at 95 °C, then 40 cycles of denaturation at 95 °C for 15 s and extension at 60 °C for 1 min. Parasite load in the lesions was calculated from the Ct values using the standard curve equation: Parasite load = 10 × [Ct − standard curve intersection)/slope]. Results are presented as the mean ± SEM of parasite concentration per lesion.

### 4.5. Gel Preparation and Storage Conditions

Carbopol-based gels of MTF (G-MTF) at concentrations of 0, 0.06, 0.17, 0.5, 1.0, and 1.5% *w*/*w* were prepared as per Neira et al. (2019) [13]. Briefly, 1% carbopol and 0.1% preservatives were dispersed in deionised water. Miltefosine was dissolved in 10% DMSO, added to the remaining water, and then mixed with the first solution under stirring. The solution was neutralised dropwise with triethanolamine until it became transparent and semi-solid. The formulations were stored at 4 °C, protected from light, in amber glass jars sealed with paraffin tape to prevent desiccation. For stability studies, the samples were incubated at 8 °C, 28 °C, 35 °C, and 45 °C under 70% relative humidity, with environmental chambers (HCP50, Memmert GmbH, Schwabach, Germany) kept in the dark. Two samples from each batch were stored and evaluated at 0, 1, 3, and 6 months.

### 4.6. Physicochemical Evaluation of Gels Stored Under Different Conditions

This experiment investigated how storing the G-MTFs at various temperatures and for different durations affects their physicochemical characteristics. The samples were visually inspected for colour and consistency. The pH level was measured with an electrochemical pH meter (Ohaus Corporation, Parsippany, NJ, USA). The pH value for each sample was measured three times. Spreadability (SP) was tested using the glass slide method. Next, 100 mg of carbopol-based gel was placed between two glass slides, and a fixed weight of 21 g was applied for 3 min. After removing the weight, the spread gel was measured using a digital calliper (Traceable^®^, VWR International, LLC, Radnor, PA, USA). The spread area (mm^2^) was calculated from the measured diameters (d) using the formula: Area = π (d/2)^2^. Results were compared to the samples just prepared. Viscosity (V) was measured with a VISCO™ viscometer (Atago Co., Ltd., Minato-ku, Tokyo, Japan) using spindle No. 4 at 3 rpm and 25 ± 0.5 °C and reported in Pa.s. Swelling (SW) was determined using the Sieve filtration method with modifications: a 1–2 g G-MTF sample was weighed (m0), and then 10 mL of distilled water at a specific pH was added. After filtering, drying for 1 h and reweighing (mt), SW percentage (SW%) was calculated at 240 min using the equation: SW% = ((m^t − m0)/m0) × 100, where mt is the mass at 240 min, and m0 is the dry sample before immersion [54]. Gel degradation was assessed under force degradation conditions, including basic hydrolysis (0.1 N NaOH), acidic hydrolysis (0.1 N HCl), and oxidative stress (3% H_2_O_2_), by analysing organoleptic changes as an initial indicator of physicochemical instability [55]. Mass loss over time was measured gravimetrically using standard pharmaceutical methods [33]. Results were expressed as percentage weight loss by dividing the mass loss (initial mass – final mass) by the initial mass (mg), then multiplying by 100.

### 4.7. Mass Spectrometry Analysis of Apparent MTF Content During Storage

Pharmaceutical-grade MTF and lyophilised samples (0.6–1.0 g) of freshly prepared G-MTF 1.0% and 1.5% formulations, as well as formulations stored at 8, 28, 35, and 45 °C (70% relative humidity) for months (Appendix A), were analysed by matrix-assisted laser desorption/ionization time-of-flight/time-of-flight mass spectrometry tandem TOF (MALDI-TOF/TOF MS) operating in MALDI-TOF (MS) mode. Samples were mixed with 500 µL α-cyano-4-hydroxycinnamic acid matrix (HCCA; Merck, Darmstadt, Germany). Analysis was performed using an UltrafleXtreme MALDI-TOF/TOF mass spectrometer (Bruker Daltonics, Bremen, Germany) equipped with a Smartbeam-II solid-state laser and operated in positive-ion reflector mode. The protonated molecular ion of MTF ([M + H]^+^, *m*/*z* 408.3) was monitored, and a calibration curve was generated using MTF standards ranging from 1 to 10 mg. Mass spectra were acquired using FlexControl 3.3 and processed and visualised using FlexAnalysis 3.3 (Flex software, version 1.3; Bruker Daltonics). Peak integration, calibration curve fitting, statistical analyses, and graphical representation were performed using OriginPro 2026b (OriginLab Corporation, Northampton, MA, USA).

### 4.8. Scanning Electron Microscopy (SEM)

Freshly prepared 0.5% MTF carbopol-based gels and their corresponding vehicle (without MTF), as well as formulations stored for 4 months at 8, 28, and 35 °C, were dried under ambient conditions, mounted on metal stubs with carbon adhesive tape, and then gold-coated. Images were acquired under high vacuum at an accelerating voltage of 15 kV using a Quanta FEG 650 field-emission scanning electron microscope (FEG-SEM). Secondary electron images were obtained with an Everhart–Thornley detector (ETD), and backscattered electron images were acquired with a solid-state backscattered electron detector (BSED/SSD).

### 4.9. Sterility Test

Gels (1 g) were diluted in 9 mL of sterile Brain Heart Infusion (BHI) broth (Merck, Rahway, NJ, USA) and incubated overnight at 37 °C or at 25 °C. Following incubation, 1:10 serial dilutions were prepared in BHI, then plated on nutrient agar for bacterial detection at 37 °C for 48 h and on Sabouraud agar for fungi and yeasts at 25 °C for 5 days. Results are expressed as colony-forming units per gram of carbopol-based gels (CFU/g).

### 4.10. Antileishmanial and Cytotoxic Activities of G-MTF Stored Under Different Conditions

Promastigotes (1 × 10^6^ parasites/mL) or PMA-differentiated THP-1 cells were treated with G-MTFs at a 1:3 dilution for 72 h at 27 °C or 32 °C, respectively; controls received no drug. Parasite growth inhibition was measured using the resazurin reduction test. A total of 20 μL of resazurin (0.11 mg/mL) was added to each well, and after 4 h, the OD was measured at 570 and 600 nm using a Synergy H1 microplate reader (BioTek Instruments, Inc., Winooski, VT, USA). The toxicity of THP-1 cells was evaluated using the MTT assay. Next, 20 µL of MTT solution (2.5 mg/mL) was added to each well for 4 h. The supernatant was removed, and insoluble formazan crystals were dissolved in 100 μL of DMSO. Absorbances were determined at 580 nm. Percentages of parasite inhibition or cell toxicity were calculated using the following formula: % parasite inhibition/% cell toxicity = 100 × (OD control group − OD treated group)/OD control group. For intracellular amastigotes, infected THP-1 cells were treated with the drug for 3 days at 32 °C, and growth inhibition was assessed microscopically by counting infected cells among 300 Giemsa-stained cells. IC_50_ or CC_50_ values were determined by nonlinear regression using Msxlfit™ software (Version 5.5, ID Business Solutions, Guildford, UK). The 50% cytotoxic concentration (CC_50_) was determined using GraphPad Software, version 8.0 (2018). Three experiments were performed. The selectivity indices (SIs) were calculated as the ratio of the drug’s activity against cells to its activity against parasites (CC_50_/IC_50_) [13,14].

### 4.11. In Vivo Efficacy of G-MTF After Storage

This experiment investigated how storing the G-MTF at various temperatures (freshly prepared, 8 °C, 28 °C, 35 °C, 45 °C) and for different durations (1, 3, 6 months) affects lesion size and parasite load in mice with CL. All conditions were tested using G-MTF at 0.5%, 1.0%, and 1.5%. A total of 131 BALB/c-infected mice were randomly assigned to 44 experimental groups, as detailed in Table 1. Additionally, groups 40 and 41 received 2.0% G-MTF, while groups 42, 43, and 44 received the gel without MTF. Infection, topical treatment for 25 days, and a 60-day follow-up were performed as described previously. The appropriate sample size (n) for our study was determined using G Power software (Statistical Power Analyses 3.1.9.4 for Windows), based on previously obtained values from our lab, with a power of 80% and alpha of 0.05. The LS reduction percentage was calculated as follows: (LS0 − LSx)/LS0 × 100, where LS0 = Initial lesion size and LSx = lesion size at each evaluation day. The study recorded the appearance of the first lesion and its duration until a well-defined CL lesion developed.

### 4.12. Dose–Response Experiments on Mice

The study examined how different G-MTF concentrations affect lesion size and parasite load. BALB/c-infected mice were randomly assigned to seven experimental groups, as shown in Table 2. Based on lesion area (mm^2^) and parasite loads at each MTF dose, the effective MTF concentration at 50 and 90% (ED_50_ and ED_90_) was calculated using sigmoidal regression with Msxlfit^TM^ software ((Version 5.5, ID Business Solutions, Guildford, UK). Concentration-response curves and EC_50_/EC_90_ values were generated during and after treatment.

## Figures and Tables

**Table 1:** In vivo antileishmanial activity of 0, 0.5, 1.0, 1.5 and 2.0% miltefosine carbopol-based gels (G-MTF) (in bold) after storage. The table presents the BALB/c mouse experimental groups (groups 1–44; n = 3) and the storage conditions of G-MTF at 0, 8, 28, 35, and 45 °C, assessed at different time points, including freshly prepared formulation and after 1, 3, and 6 months of storage.

	0.5% G-MTF				1.0% G-MTF				
°C	Freshly Prepared	1 m	3 m	6 m	Freshly Prepared	1 m	3 m	6 m	
0	1	ND ^1^	ND	ND	14	ND	ND	ND	
8		2	3	4		15	16	17	
28		5	6	7		18	19	20	
35		8	9	10		21	22	23	
45		11	12	13		24	25	26	
	**1.5%**				**2.0%**				**0%**
°C	Freshly Prepared	1 m	3 m	6 m	Freshly prepared	1 m	3 m	6 m	
0	27	ND	ND	ND	40				42
8		28	29	30					
28		31	32	33		41			43
35		34	35	36					
45		37	38	39					44

^1^ Not determined.

**Table 2:** Experimental groups and treatment protocol of dose–response experiments.

Group	Mice (n)	G-MTF ^1^ (%)	MTF (mM Dose)	MTF (mM Final Dose)	MTF (mg/kg/ Dose)	Way	Doses (n)	Follow Up (60 Days)
1	6	0	0	0	0	Topic	25	60
2	8	0.06	1.47	36.8	1.2	Topic	25	60
3	8	0.17	4.17	104.2	3.4	Topic	25	60
4	6	0.50	12.26	902.1	10	Topic	25	60
5	8	1.50	36.80	306.5	30	Topic	25	60
6	6	MTF solution	30 mg/kg	450 mg/kg	30	Oral	15	60
7	6	ND ^2^	-	-	-	-	-	60

^1^ MTF obtained from Gold Biotechnology^®^; ^2^ Not determined.

## Data Availability

The data presented in this study are openly available in the article.

## Supplementary Materials

The following supporting information can be downloaded at https://www.mdpi.com/article/10.3390/gels12090779/s1. Figure S1: Scanning electron microscopy images of 0.5% MTF carbopol-based gel under different storage conditions; Figure S2: Influence of storage temperature (8, 28, 35, and 45 °C) and incubation time (1−6 months) on the in vitro parasite and cell activities; Figure S3: Time-dependent dose–response analysis of G-MTF in BALB/c mice with CL. Table S1: Effect of storage conditions on the colour and consistency of G-MTF formulations; Table S2: Effect of storage conditions on the pH of G-MTF formulations; Table S3: Effect of storage conditions on the spreadability of G-MTF formulations; Table S4: Effect of storage conditions on the viscosity of G-MTF formulations; Table S5: Effect of storage conditions on the swelling of G-MTF formulations; Table S6: Effect of storage conditions on the degradation of G-MTF formulation. Table S7: Effect of storage conditions on the mass loss of G-MTF formulation; Table S8: Effect of storage conditions on the antileishmanial activity of G-MTF against *L. amazonensis* promastigotes; Table S9: Effect of storage conditions on the antileishmanial activity of G-MTF against *L. amazonensis* intracellular amastigotes; Table S10: Effect of storage conditions on the cytotoxic activity of G-MTF against THP-1 cells. Table S11: Selectivity index (SI), calculated as CC_50_/IC_50_ (promastigotes).

## Abbreviations

The following abbreviations are used in this manuscript:

CL	Cutaneous leishmaniasis
DMSO	Dimethyl sulfoxide
EC_50_	Effective concentration 50
ED_50_	Effective dose 50
G-MTF	Miltefosine carbopol-based gels
HCCA	Cyano-4-hydroxycinnamic acid matrix
LS	Lesion size
MTF	Miltefosine
SP	Spreadability
SW	Swelling
V	Viscosity

## Appendix A

**Table A1:** Comparative table with other methodologies used for miltefosine quantification [39,40,41].

Parameter	UIS MALDI-TOF MS Mode	Van Kampen et al. (2006)	Kip et al. (2015) and Dorlo	Jaiswal et al. (2016)
Aim	To evaluate the apparent MTF content during MTF carbopol-based gels storage	To develop a quantitative MALDI-TOF method for low molecular weight drugs and to demonstrate its application in biological samples	Quantify intracellular MTF for pharmacokinetic studies	Quantify MTF in human and hamster plasma for pharmacokinetic studies
Analyte(s)	Miltefosine	Lopinavir, ritonavir, and 24 other drugs	Miltefosine	Miltefosine
Biological/ pharmaceutical matrix	Carbopol® hydrogel.	Human PBMCs	Human PBMCs	Human plasma and hamster plasma
Application	Quality control and pharmaceutical stability	Methodological development for quantitative analysis of small molecules using MALDI-TOF	Intracellular pharmacokinetics	Systemic pharmacokinetics
Sample preparation	Freeze-drying and co-crystallisation with HCCA	Solid phase extraction (SPE), pipetting robot and crystallisation on AnchorChip®	Cell lysis + SPE	Protein precipitation
Analytical technique	MALDI-TOF/TOF MS operating in MALDI-TOF (MS) mode	MALDI-TOF MS.	LC-MS/MS	LC-ESI-MS/MS
Ionization source	HCCA as a matrix for ionisation	F20TPP as matrix	Electrospray (ESI)	Positive electrospray (ESI)
Mode of acquisition	Positive reflector.	Positive reflector	MRM	MRM
Internal standard	Pharmaceutical grade miltefosine	Indinavir or saquinavir according to the experiment	Miltefosine-D4.	Phenacetin
Calibration curve	1–10 mg.	Weighted square fit (1/x^2^).	4–2000 ng/mL	2.5–400 ng/mL
LLOQ	Not determined (semi-quantitative method)	25 fmol in PBMC extracts; up to 1 fmol for pure lopinavir	4 ng/mL	2.5 ng/mL
Key benefits	Rapid analysis without chromatographic separation; Suitable for formulation stability monitoring	High throughput (≈2 min per sample), minimal chemical interference using F20TPP, and reproducible quantification of small molecules using MALDI-TOF	High sensitivity and specificity for cell studies	High sensitivity for plasma pharmacokinetics
Limitations	Method oriented to structural confirmation and estimation of content; not validated for pharmacokinetics	Limited dynamic range compared to LC-MS/MS and need to optimise matrix and data processing for good reproducibility	Requires specialised chromatographic instrumentation	Requires sample preparation and specialised chromatographic separation

HCCA matrix: α-cyano-4-hydroxycinnamic acid; F20TPP: meso-tetrakis (pentafluorophenyl) porphyrin matrix; PBMCs: peripheral blood mononuclear cells; MALDI-TOF MS: matrix-assisted laser desorption/ionization time-of-flight mass spectrometry, single TOF; MALDI-TOF/TOF MS: matrix-assisted laser desorption/ionization time-of-flight/time-of-flight mass spectrometry tandem TOF; LC-ESI-MS/MS: liquid chromatography-electrospray ionization tandem mass spectrometry); LC-MS/MS: liquid chromatography-tandem mass spectrometry.

## References

[1] World Health Organization (WHO) Fact Sheets/Detail/Leishmaniasis. https://www.who.int/news-room/fact-sheets/detail/leishmaniasis.

[2] Azim M., Khan S.A., Ullah S., Ullah S., Anjum S.I. (2021). Therapeutic Advances in the Topical Treatment of Cutaneous Leishmaniasis: A Review. PLoS Negl. Trop. Dis..

[3] Pan American Health Organization (2022). Guideline for the Treatment of Leishmaniasis in the Americas.

[4] Carnielli J.B.T., Monti-Rocha R., Costa D.L., Sesana A.M., Pansini L.N.N., Segatto M., Mottram J.C., Costa C.H.N., Carvalho S.F.G., Dietze R. (2019). Natural Resistance of Leishmania Infantum to Miltefosine Contributes to the Low Efficacy in the Treatment of Visceral Leishmaniasis in Brazil. Am. J. Trop. Med. Hyg..

[5] Mendes L., Guerra J.O., Costa B., da Silva A.S., Guerra M.d.G.B., Ortiz J., Doria S.S., da Silva G.V., de Jesus D.V., Barral-Netto M. (2021). Association of Miltefosine with Granulocyte and Macrophage Colony-Stimulating Factor (GM-CSF) in the Treatment of Cutaneous Leishmaniasis in the Amazon Region: A Randomized and Controlled Trial. Int. J. Infect. Dis..

[6] Rijal S., Ostyn B., Uranw S., Rai K., Bhattarai N.R., Dorlo T.P.C., Beijnen J.H., Vanaerschot M., Decuypere S., Dhakal S.S. (2013). Increasing Failure of Miltefosine in the Treatment of Kala-Azar in Nepal and the Potential Role of Parasite Drug Resistance, Reinfection, or Noncompliance. Clin. Infect. Dis..

[7] Del Mar Castro M., Gomez M.A., Kip A.E., Cossio A., Ortiz E., Navas A., Dorlo T.P.C., Saravia N.G. (2017). Pharmacokinetics of Miltefosine in Children and Adults with Cutaneous Leishmaniasis. Antimicrob. Agents Chemother..

[8] Hall A.V., Gostick I.E.F., Yufit D.S., Marchant G.Y., Kirubakaran P., Madu S.J., Li M., Steel P.G., Steed J.W. (2022). Integral Role of Water in the Solid-State Behavior of the Antileishmanial Drug Miltefosine. Cryst. Growth Des..

[9] Dorlo T.P.C., Balasegaram M., Beijnen J.H., de Vries P.J. (2012). Miltefosine: A Review of Its Pharmacology and Therapeutic Efficacy in the Treatment of Leishmaniasis. J. Antimicrob. Chemother..

[10] Palić S., Beijnen J.H., Dorlo T.P.C. (2022). An Update on the Clinical Pharmacology of Miltefosine in the Treatment of Leishmaniasis. Int. J. Antimicrob. Agents.

[11] Ribeiro L.R., Silva S.N., Saliba M.F., de Pina Carvalho J., Cota G. (2024). Safety Profile of Miltefosine in the Treatment of Cutaneous Leishmaniasis. PLoS ONE.

[12] Ware J.A.M., O’Connell E.M., Brown T., Wetzler L., Talaat K.R., Nutman T.B., Nash T.E. (2021). Efficacy and Tolerability of Miltefosine in the Treatment of Cutaneous Leishmaniasis. Clin. Infect. Dis..

[13] Neira L.F., Mantilla J.C., Escobar P. (2019). Anti-Leishmanial Activity of a Topical Miltefosine Gel in Experimental Models of New World Cutaneous Leishmaniasis. J. Antimicrob. Chemother..

[14] Neira L.F., Mantilla J.C., Escobar P. (2023). Monitoring Cutaneous Leishmaniasis Lesions in Mice Undergoing Topical Miltefosine Treatment. Sci. Pharm..

[15] Li J., Mooney D.J. (2016). Designing Hydrogels for Controlled Drug Delivery. Nat. Rev. Mater..

[16] Hu X., Wei W., Qi X., Yu H., Feng L., Li J., Wang S., Zhang J., Dong W. (2015). Preparation and Characterization of a Novel PH-Sensitive Salecan-g-Poly(Acrylic Acid) Hydrogel for Controlled Release of Doxorubicin. J. Mater. Chem. B.

[17] Yu H., Qi X., Chen Y., Chen Y., Zhang J., Dong W., Shen J., Jiang T. (2025). A Procyanidin/Fe3O4 Nanoparticle-Reinforced Pullulan/Chitosan Hydrogel for Treating Diabetic Wounds. Chin. Chem. Lett..

[18] Merino-Gómez M., Godoy-Gallardo M., Wendner M., Mateos-Timoneda M.A., Gil F.J., Perez R.A. (2023). Optimization of Guanosine-Based Hydrogels with Boric Acid Derivatives for Enhanced Long-Term Stability and Cell Survival. Front. Bioeng. Biotechnol..

[19] Szymańska E., Czajkowska-Kośnik A., Winnicka K. (2018). Comparison of Rheological, Drug Release, and Mucoadhesive Characteristics upon Storage between Hydrogels with Unmodified or Beta-Glycerophosphate-Crosslinked Chitosan. Int. J. Polym. Sci..

[20] Peralta M.F., Usseglio N.A., Bracamonte M.E., Guzmán M.L., Olivera M.E., Marco J.D., Barroso P.A., Carrer D.C. (2022). Efficacy of Topical Miltefosine Formulations in an Experimental Model of Cutaneous Leishmaniasis. Drug Deliv. Transl. Res..

[21] Van Bocxlaer K., Yardley V., Murdan S., Croft S.L. (2016). Topical Formulations of Miltefosine for Cutaneous Leishmaniasis in a BALB/c Mouse Model. J. Pharm. Pharmacol..

[22] Hernandez I.P., Montanari J.A.M., Rivero P.E. (2014). In Vitro Activity against Leishmania and Human Skin Permeation of Miltefosine Ultradeformable Liposomes. Rev. Cuba. Med. Trop..

[23] Van Bocxlaer K., Croft S.L. (2021). Pharmacokinetics and Pharmacodynamics in the Treatment of Cutaneous Leishmaniasis - Challenges and Opportunities. RSC Med. Chem..

[24] Wijnant G.J., Van Bocxlaer K., Francisco A.F., Yardley V., Harris A., Alavijeh M., Murdan S., Croft S.L. (2018). Local Skin Inflammation in Cutaneous Leishmaniasis as a Source of Variable Pharmacokinetics and Therapeutic Efficacy of Liposomal Amphotericin B. Antimicrob. Agents Chemother..

[25] Lubrizol Advanced Materials (2023). Carbopol® Ultrez 10 NF Polymer Technical Data Sheet.

[26] Islam M.T., Rodríguez-Hornedo N., Ciotti S., Ackermann C. (2004). Rheological Characterization of Topical Carbomer Gels Neutralized to Different PH. Pharm. Res..

[27] WHO (2021). Stability Testing of Active Pharmaceutical Ingredients and Finished Pharmaceutical Products: Stability Conditions for WHO Member States by Region.

[28] Weber E., Moyers-González M., Burghelea T.I. (2012). Thermorheological Properties of a Carbopol Gel under Shear. J. Nonnewton. Fluid Mech..

[29] Lambers H., Piessens S., Bloem A., Pronk H., Finkel P. (2006). Natural Skin Surface PH Is on Average below 5, Which Is Beneficial for Its Resident Flora. Int. J. Cosmet. Sci..

[30] Stojkov G., Niyazov Z., Picchioni F., Bose R.K. (2021). Relationship between Structure and Rheology of Hydrogels for Various Applications. Gels.

[31] Fresno Contreras M.J., Ramírez Diéguez A., Jiménez Soriano M.M. (2001). Viscosity and Temperature Relationship in Ethanol/Water Mixtures Gelified with Carbopol® Ultrez^TM^ 10. Farmaco.

[32] Rowe R.C., Sheskey P.J., Quinn M. (2005). Handbook of Pharmaceutical Excipients.

[33] (2003). International Council for Harmonisation (ICH). Q1A(R2): Stability testing of new drug substances and products: Guidance for industry 2003. SubStance.

[34] Hoare T.R., Kohane D.S. (2008). Hydrogels in Drug Delivery: Progress and Challenges. Polymer.

[35] United States Pharmacopeial Convention (2026). <51>Antimicrobial Effectiveness Testing. United States Pharmacopeia and National Formulary (USP-NF).

[36] Duncan M.W., Roder H., Hunsucker S.W. (2008). Quantitative Matrix-Assisted Laser Desorption/Ionization Mass Spectrometry. Brief. Funct. Genom. Proteom..

[37] Wang C.C., Lai Y.H., Ou Y.M., Chang H.T., Wang Y.S. (2016). Critical Factors Determining the Quantification Capability of Matrix-Assisted Laser Desorption/Ionization-Time-of-Flight Mass Spectrometry. Philos. Trans. R. Soc. A Math. Phys. Eng. Sci..

[38] Wang P., Giese R.W. (2017). Recommendations for Quantitative Analysis of Small Molecules by Matrix-Assisted Laser Desorption Ionization Mass Spectrometry. J. Chromatogr. A.

[39] Jaiswal S., Sharma A., Shukla M., Lal J. (2016). LC-Coupled ESI MS for Quantification of Miltefosine in Human and Hamster Plasma. Bioanalysis.

[40] Kip A.E., Rosing H., Hillebrand M.J.X., Castro M.M., Gomez M.A., Schellens J.H.M., Beijnen J.H., Dorlo T.P.C. (2015). Quantification of Miltefosine in Peripheral Blood Mononuclear Cells by High-Performance Liquid Chromatography-Tandem Mass Spectrometry. J. Chromatogr. B Anal. Technol. Biomed. Life Sci..

[41] Van Kampen J.J.A., Burgers P.C., De Groot R., Luider T.M. (2006). Qualitative and Quantitative Analysis of Pharmaceutical Compounds by MALDI-TOF Mass Spectrometry. Anal. Chem..

[42] World Health Organization (2025). Safe Management of Pharmaceutical Waste from Health Care Facilities: Global Best Practices.

[43] Boxall A.B.A., Rudd M.A., Brooks B.W., Caldwell D.J., Choi K., Hickmann S., Innes E., Ostapyk K., Staveley J.P., Verslycke T. (2012). Pharmaceuticals and Personal Care Products in the Environment: What Are the Big Questions?. Environ. Health Perspect..

[44] Yardley V., Croft S.L., De Doncker S., Dujardin J.C., Koirala S., Rijal S., Miranda C., Llanos-Cuentas A., Chappuis F. (2005). The Sensitivity of Clinical Isolates of Leishmania from Peru and Nepal to Miltefosine. Am. J. Trop. Med. Hyg..

[45] Lessa V.L., Gonçalves G., Santos B., Cavalari V.C., da Costa Vieira R.F., Figueiredo F.B. (2024). In Vitro Evaluation of the Combinatorial Effect of Naringenin and Miltefosine against Leishmania Amazonensis. Pharmaceuticals.

[46] Neira L.F., Costa-Silva H.M., Ribeiro J.M., Murta S.M.F., Escobar P. (2025). Biological and Molecular Characterisation of in Vitro Selected Miltefosine-Resistant Leishmania Amazonensis Lines. Int. J. Parasitol. Drugs Drug Resist..

[47] Kavian Z., Alavizadeh S.H., Golmohamadzadeh S., Badiee A., Khamesipour A., Jaafari M.R. (2019). Development of Topical Liposomes Containing Miltefosine for the Treatment of Leishmania Major Infection in Susceptible BALB/c Mice. Acta Trop..

[48] Williams A.C., Barry B.W. (2004). Penetration Enhancers. Adv. Drug Deliv. Rev..

[49] Rakotomanga M., Blanc S., Gaudin K., Chaminade P., Loiseau P.M. (2007). Miltefosine Affects Lipid Metabolism in Leishmania Donovani Promastigotes. Antimicrob. Agents Chemother..

[50] El-On J., Jacobs G.P., Witztum E., Greenblatt C.L. (1984). Development of Topical Treatment for Cutaneous Leishmaniasis Caused by Leishmania Major in Experimental Animals. Antimicrob. Agents Chemother..

[51] Escobar P., Yardley V., Croft S.L. (2001). Activities of Hexadecylphosphocholine (Miltefosine), Ambisome, and Sodium Stibogluconate (Pentostam) against Leishmania Donovani in Immunodeficient Scid Mice. Antimicrob. Agents Chemother..

[52] Sundar S., Chakravarty J. (2015). An Update on Pharmacotherapy for Leishmaniasis. Expert Opin. Pharmacother..

[53] Monroy-Ostria A., Nasereddin A., Monteon V.M., Guzmán-Bracho C., Jaffe C.L. (2014). ITS1 PCR-RFLP Diagnosis and Characterization of Leishmania in Clinical Samples and Strains from Cases of Human Cutaneous Leishmaniasis in States of the Mexican Southeast. Interdiscip. Perspect. Infect. Dis..

[54] Zhang K., Feng W., Jin C. (2020). Protocol Efficiently Measuring the Swelling Rate of Hydrogels. MethodsX.

[55] Bakshi M., Singh S. (2002). Development of Validated Stability-Indicating Assay Methods—Critical Review. J. Pharm. Biomed. Anal..

