# Supplementary material for: Optimisation of a Topical Miltefosine Carbopol-Based Gel for Cutaneous Leishmaniasis: Antileishmanial Activity After Storage and Drug Response
Source: Gels. 2026 Aug 31;12(9):779. doi: 10.3390/gels12090779 (PMC13606578; doi:10.3390/gels12090779)

Article

Optimisation of a Topical Miltefosine Carbopol-Based Gel for Cutaneous Leishmaniasis: Antileishmanial Activity After Storage and Drug Response

Diana Paola Peña *, Laura F. Neira and Patricia Escobar *

Table S1. Effect of storage conditions on the colour and consistency of G-MTF formulations. Colour and consistency of 0, 0.5, 1.0, 1.5 and 2.0% miltefosine carbopol-based gels (G-MTF) (in bold), freshly prepared and after 1, 3, and 6 months (m) of incubation at 8, 28, 35, and 45 °C. Changes observed are shown in red.

	**0.5% G-MTF**				**1.0 % G-MTF**				
°C	Just prepared	1 m	3 m	6 m	Just prepared	1 m	3 m	6 m	
0	T/SS	NA	NA	NA	T/SS	NA	NA	NA	
8	NA	T/SS	T/SS	T/**S**	NA	T/SS	T/ SS	**T/S**	
28	NA	T/SS	T/SS	T/SS	NA	T/SS	T/ SS	T/SS	
35	NA	T/SS	T/SS	T/SS	NA	T/SS	T/SS	T/SS	
45	NA	T/SS	T/SS	**T/ L**	NA	T/SS	T/SS	**T/L**	
	**1.5% G-MTF**				**2.0% G-MTF**				**0% G-MTF**
°C	Just prepared	1 m	3 m	6 m	Just prepared	1 m	3 m	6 m	
0	W/SS	NA	NA	NA	W/SS	NA	NA	NA	T/SS
8	NA	W/SS	W/SS	**T/S**	NA	W/SS	W/SS	ND	
28	NA	W/SS	T/SS	T/SS	NA	W/SS	W/SS	ND	
35	NA	T/SS	T/SS	T/SS	NA	W/SS	T/SS	ND	
45	NA	T/SS	T/SS	**T/L**	NA	W/SS	T/SS	ND	

T: transparent, S: solid, SS: semi-solid, W: off-white (whitish), L: liquid. NA: Not applicable

Table S2. Effect of storage conditions on the pH of 0, 0.5, 1.0, 1.5 and 2.0% miltefosine carbopol-based gels (G-MTF) (in bold). The results were expressed as the median of pH values ± SD. Each sample was tested in triplicate.

	**0.5% G-MTF**				**1.0 % G-MTF**				
°C	Just prepared	1 m	3 m	6 m	Just prepared	1 m	3 m	6 m	
0	5.93±0.1	NA	NA	NA	6.13±0.2	NA	NA	NA	
8	NA	5.90±0.1	6.07±0.2	5.83±0.2	NA	6.20±0.1	6.20 ± 0.1	5.93±0.2	
28	NA	6.07±0.1	5.83±0.2	5.93±0.1	NA	6.2± 0.1	5.93±0.2	6.13±0.2	
35	NA	6.03±0.2	6.10±0.1	6.03±0.2	NA	6.07±0.2	6.27 ±0.1	6.23±0.1	
45	NA	5.87±0.2	6.03±0.1	5.93±0.1	NA	6.27±0.1	6.20±0.12	6.20±0.2	
	**1.5% G-MTF**				**2.0% G-MTF**				**0% G-MTF**
°C	Just prepared	1 m	3 m	6 m	Just prepared	1 m	3 m	6 m	
0	6.33±0.1	NA	NA	NA	6.17±0.2	NA	NA	NA	6.07±0.2
8	NA	6.23±0.1	6.20±0.1	6.27±0.1	NA	5.97±0.6	6.03±0.1	ND	
28	NA	6.33±0.2	6.28±0.1	6.33±0.2	NA	6.03±0.1	6.05±0.1	ND	
35	NA	6.23±0.1	6.27±0.1	6.25±0.1	NA	5.93±0.1	6.10±0.1	ND	
45	NA	6.23±0.2	6.17±0.2	6.20±0.1	NA	5.95±0.1	5.97±0.1	ND	

Table S3. Effect of storage conditions on the spreadability of 0, 0.5, 1.0, 1.5 and 2.0% miltefosine carbopol-based gels (G-MTF) (in bold). It was measured by the glass slide method. The area of spread was expressed in mm². The results were expressed as the median (mm²) ± SD. Each sample was tested in triplicate. Significant differences between storage times were determined using repeated-measures ANOVA, followed by Dunnett's multiple-comparisons test. Statistical significance levels are indicated as $p < 0.05$ (*), $p < 0.01$ (**), and $p < 0.001$ (***).

	**0.5% G-MTF**				**1.0 % G-MTF**				
°C	Just prepared	1 m	3 m	6 m	Just prepared	1 m	3 m	6 m	
0	141.4±0.9	NA	NA	NA	133.1±0.6	NA	NA	NA	
8	NA	91.0±15.6	78.7±3.7*	82.0 ±4.1*	NA	84.2±0.5	70.0±11.3*	75.7±6.8*	
28	NA	139.3±6.8	143.7±9.8	150.0±6.8	NA	137.1±5.1	135.6±7.2	132.2±7.9	
35	NA	162.4±10.4	174.6±3.7*	188.0±4.3*	NA	166.8±5.9	182.7±10.2*	192.9±1.8**	
45	NA	167.8±2.3	185.7±4.3*	207.5±14.4**	NA	147.9±8.4	190.7±8.1**	196.0±7.8**	

	**1.5% G-MTF**				**2.0% G-MTF**				**0% G-MTF**
°C	Just prepared	1 m	3 m	6 m	Just prepared	1 m	3 m	6 m	
0	137.1±1.9	NA	NA	NA	144.1±9.2	NA	NA	NA	136.9±6.8
8	NA	106.8±168	100.9±23.1	99.2±7.9	NA	110.8±9.9	88.9±1.9*	ND	
28	NA	151.0±5.3	147.9±4.6	160.6±9.6*	NA	155.7±6.3	153.0±4.8	ND	
35	NA	157.9±3.2	178.4±12.8*	181.4±8.5**	NA	149.7±11.3	173.8±8.8*	ND	
45	NA	151.2±2.3	181.8±7.6**	208.2±11.3***	NA	165.4±6.9	183.1±11.0**	ND	

Table S4. Effect of storage conditions on the viscosity of 0, 0.5, 1.0, 1.5 and 2.0% miltefosine carbopol-based gels (G-MTF) (in bold). It was measured with a VISCO™ viscometer. The results were expressed as the median (Pa*s) ± SD. Each sample was tested in triplicate. Significant differences between storage times were determined using repeated-measures ANOVA, followed by Dunnett's multiple-comparisons test. Statistical significance levels are indicated as $p < 0.05$ (*), $p < 0.01$ (**), and $p < 0.001$ (***).

	**0.5% G-MTF**				**1.0% G-MTF**				
°C	Just prepared	1 m	3 m	6 m	Just prepared	1 m	3 m	6 m	
0	113.±2.3	NA	NA	NA	112.0±13.3	NA	NA	NA	
8	NA	115.2±5.9	168.1±2.6**	167.7±5.5**	NA	123.9±8.2	167.4±15.2**	167.3±5.6**	
28	NA	150.5±10.6*	144.1±7.1	127.2±3.3	NA	98.6±5.1	142.8±6.8*	126.7±4.3	
35	NA	111.8±10.5	98.0±4.2*	97.3±3.5*	NA	109.4±14.7	93.8±0.5*	89.8±2.2*	
45	NA	106.0±3.2	92.3±4.9*	95.6±2.9*	NA	113.6±1.1	92.8±5.0*	90.2±3.3*	
	**1.5% G-MTF**				**2.0% G-MTF**				**0% G-MTF**
°C	Just prepared	1 m	3 m	6 m	Just prepared	1 m	3 m	6 m	
0	114.3±1.2	NA	NA	NA	124.1±3.6	NA	NA	NA	118.4 ± 11.3
8	NA	117.0±7.1	162.9 ± 6.4**	161.3±5.3**	NA	123.4 ± 2.2	167.2±6.5**	ND	
28	NA	115.2±5.9	109.3±11.0	115.7±7.7	NA	136.2±5.9*	123.8±7.2	ND	
35	NA	108.8±8.1	90.9±6.2	94.5±2.5*	NA	120.3±6.6	95.2±2.9*	ND	
45	NA	113.3±4.6	86.6±1.6*	88.2±3.9*	NA	116.1±2.0	95.0±2.3*	ND	

Table S5. Effect of storage conditions on the swelling of 0, 0.5, 1.0, 1.5 and 2.0% miltefosine carbopol-based gels (G-MTF) (in bold). Swelling (SW) was determined using the Sieve filtration method. Swelling capacity was expressed as the mean percentage ± SD. Significant differences between storage times were determined using repeated-measures ANOVA, followed by Dunnett's multiple-comparisons test. Statistical significance levels are indicated as $p < 0.05$ (*), $p < 0.01$ (**), and $p < 0.001$ (***).

	**0.5% G-MTF**				**1.0% G-MTF**			
°C	Just prepared	1 m	3 m	6 m	Just prepared	1 m	3 m	6 m
0	23.8±6.5	NA	NA	NA	20.6±3.5	NA	NA	NA
8	NA	17.9±5.1	21.3 ± 8.4	22.0±7.5	NA	28.6±10.1	23.6±2.0	19.5±14.8
28	NA	32.4±7.2	22.7±10.3	23.6±5.1	NA	24.1±8.4	38.9±7.9	31.8±6.4
35	NA	27.9±0.9	42.9±10.1*	45.8±0.5*	NA	29.2±5.9	32.1± 1.8	47.8±8.6*
45	NA	43.1±4.4	53.8±10.9*	65.3±33.4**	NA	15.0±2.4	50.0±4.7**	73.6±21.6***

	**1.5% G-MTF**				**2.0% G-MTF**				**0% G-MTF**
°C	Just prepared	1 m	3 m	6 m	Just prepared	1 m	3 m	6 m	
0	23.0±10.9	NA	NA	NA	33.3±7.1	NA	NA	NA	24.3±2.4
8	NA	15.6±4.4	23.3±4.7	11.9 ± 5.0	NA	17.6±0.3	19.2±8.2	ND	
28	NA	20.6±3.5	26.1±1.6	43.2±9.6	NA	26.1±1.6	24.1±8.4	ND	
35	NA	24.7±6.7	40.6±0.8	43.2±9.6	NA	30.0±4.7	36.7±4.7	ND	
45	NA	17.9±5.1	60.7±15.2**	60.3±9.1**	NA	36.1±13.3	58.3±11.8**	ND	

Table S6. Effect of storage conditions on the degradation of 0, 0.5, 1.0, 1.5 and 2.0% miltefosine carbopol-based gels (G-MTF) (in bold). Gel degradation was assessed under forced degradation conditions. Acid solution, HCl 1N (Ac); basic solution, NaOH 1N (Bs); or oxygen peroxide 3% (Ox).

	**0.5% G-MTF**				**1.0% G-MTF**				
°C	Just prepared	1 m	3 m	6 m	Just prepared	1 m	3 m	6 m	
0	Ac/Bs	NA	NA	NA	Ac/Bs	NA	NA	NA	
8	NA	Ac/Bs	Ac/Bs	ND	NA	Ac/Bs	Ac/Bs	ND	
28	NA	Ac/Bs	Ac/Bs	ND	NA	Ac/Bs	Ac/Bs	ND	
35	NA	Ac/Bs	Ac/Bs	ND	NA	Ac/Bs	Ac/Bs	ND	
45	NA	Ac/Bs	Ac/Bs	ND	NA	Ac/Bs	Ac/Bs	ND	
	**1.5% G-MTF**				**2.0% G-MTF**				**0% G-MTF**
°C	Just prepared	1 m	3 m	6 m	Just prepared	1 m	3 m	6 m	
0	Ac/Bs	NA	NA	NA	Ac/Bs	NA	NA	NA	Ac/Bs
8	NA	Ac/Bs	Ac/Bs	ND	NA	Ac/Bs	Ac/Bs	ND	
28	NA	Ac/Bs	Ac/Bs	ND	NA	Ac/Bs	Ac/Bs	ND	
35	NA	Ac/Bs	Ac/Bs	ND	NA	Ac/Bs	Ac/Bs	ND	
45	NA	Ac/Bs	Ac/Bs	ND	NA	Ac/Bs	Ac/Bs	ND	

Table S7. Effect of storage conditions on the mass loss of 0, 0.5, 1.0, 1.5 and 2.0% miltefosine carbopol-based gels (G-MTF) (in bold). Mass loss over time was measured gravimetrically using a standard pharmaceutical method. Results were expressed as a percentage of mass loss over time (mean ± SD), n = 3. Significant differences between storage times were determined using repeated-measures ANOVA, followed by Dunnett's multiple-comparisons test. Statistical significance levels are indicated as $p < 0.05$ (*), $p < 0.01$ (**), and $p < 0.001$ (***).

	**0.5% G-MTF**				**1.0% G-MTF**			
°C	Just prepared	1 m	3 m	6 m	Just prepared	1 m	3 m	6 m
0	0	NA	NA	NA	0	NA	NA	NA
8	NA	ND	2.0±0.3	8.3±1,5	NA	ND	6.3±2.2	20.5±4.5
28	NA	ND	2.1±0.5	3.2 ± 1.1	NA	ND	1.6±0.3	2.5 ± 0.7
35	NA	ND	4.0±1.6	12.3±3.4	NA	ND	2.7±2.5	18.3±4.1*
45	NA	ND			NA	ND		

	**1.5% G-MTF**				**2.0% G-MTF**				**0% G-MTF**
°C	Just prepared	1 m	3 m	6 m	Just prepared	1 m	3 m	6 m	
0	0	NA	NA	NA	0	NA	NA	NA	6.7±3.1
8	NA	ND	5.4±2.1	11.3±2.6	NA	ND	7.2±1.5	17.6±2.7*	
28	NA	ND	3.5±1.2	5.2±1.4	NA	ND	2.1±1.6	3.0±2.6	
35	NA	ND	2.7±1.4	18.3±3.6*	NA	ND	3.7±0.7	12.3±5.2	
45	NA	ND	1.6±0.8	18.5±4.2*	NA	ND	4.5±1.3	15.3±3.6	

Table S8. Effect of storage conditions on the antileishmanial activity of 0, 0.5, 1.0, 1.5 and 2.0% miltefosine carbopol-based gels (G-MTF) (in bold) against *L. amazonensis* promastigotes. Parasite growth inhibition was measured using the resazurin reduction test. The Inhibitory concentration (IC_{50}) was calculated. Results were expressed as the mean IC_{50} ± SD of G-MTF. Each sample was tested in triplicate.

**IC_{50} (µg/µL)**									
	**0.5% G-MTF**				**1.0 % G-MTF**				
°C	Just prepared	1 m	3 m	6 m	Just prepared	1 m	3 m	6 m	
0	1.48±0.12	NA	NA	NA	0.76±0.03	NA	NA	NA	
8	NA	1.14±0.3	1.10±0.2	1.51±0.21	NA	0.75±0.02	0.66±0.03	0.76±0.02	
28	NA	1.02±0,2	1.28±0,2	1.68±0,15	NA	0.64±0.03	0.84±0.02	0.70±0.03	
35	NA	1.12±0.2	1.91±0.4	2.4±0.34	NA	0.76±0.12	0.62±0.17	1.64±0.08	
45	NA	0.97±0.2	1.88±0.1	2.12±0.26	NA	0.75±0.03	0.62±0.02	1.73±0.09	
	**1.5% G-MTF**				**2.0% G-MTF**				**0% G-MTF**
°C	Just prepared	1 m	3 m	6 m	Just prepared	1 m	3 m	6 m	
0	0.34±0.04	NA	NA	NA	0.28±0.05	NA	NA	NA	>16
8	NA	0.35±0.0	0.37±0.1	0.46±0.02	NA	0.26±0.02	0.27±0.01	ND	
28	NA	0.35±0.0	0.44±0.1	0.40±0.05	NA	0.33±0.02	0.29±0.03	ND	
35	NA	0.38±0.1	0.42±0.0	0.61±0.09	NA	0.32±0.04	0.37±0.02	ND	
45	NA	0.43±0.0	0.65±0.1	1.18±0.08	NA	0.27±0.02	0.56±0.03	ND	

Table S9. Effect of storage conditions on the antileishmanial activity of 0, 0.5, 1.0, 1.5 and 2.0% miltefosine carbopol-based gels (G-MTF) (in bold) against *L. amazonensis* intracellular amastigotes. Growth inhibition was assessed microscopically by counting infected cells among 300 Giemsa-stained cells. Results were expressed as the mean IC_{50} ± SD of G-MTF. Each sample was tested in duplicate.

					**IC_{50} (μg/μL)**				
	**0.5% G-MTF**				**1.0 % G-MTF**				
°C	Just prepared	1 m	3 m	6 m	Just prepared	1 m	3 m	6 m	
0	2.06±0.10	NA	NA	NA	1.91 ± 0.21	NA	NA	NA	
8	NA	NA	NA	2.21 ± 0.23	NA	NA	NA	2.12 ± 0.52	
28	NA	NA	NA	2.10 ± 0.17	NA	NA	NA	2.23 ± 0.27	
35	NA	NA	NA	2.36 ± 0.11	NA	NA	NA	2.28 ± 0.62	
45	NA	NA	NA	2.24 ± 0.14	NA	NA	NA	2.44 ± 0.31	
	**1.5% G-MTF**				**2.0% G-MTF**				**0% G-MTF**
°C	Just prepared	1 m	3 m	6 m	Just prepared	1 m	3 m	6 m	
0	1.58±0.20	NA	NA	NA	1.17 ± 0.27	NA	NA	NA	>16
8	NA	NA	NA	1.12 ± 0,45	NA	NA	1.24±0.25	NA	
28	NA	NA	NA	1.89 ± 0.41	NA	NA	1.26±0.31	NA	
35	NA	NA	NA	2.21 ± 0.51	NA	NA	1.32±0,21	NA	
45	NA	NA	NA	2.55 ± 0.32	NA	NA	1.53±0.61	NA	

Table S10. Effect of storage conditions on the cytotoxic activity of 0, 0.5, 1.0, 1.5 and 2.0% miltefosine carbopol-based gels (G-MTF) (in bold) against THP-1 cells. The toxicity of THP-1 cells was evaluated using the MTT assay. Results were expressed as the mean CC_{50} ± SD of G-MTF. Each sample was tested in triplicate.

**CC_{50} (μg/μL)**									
	**0.5% G-MTF**				**1.0% G-MTF**				
°C	Just prepared	1 m	3 m	6 m	Just prepared	1 m	3 m	6 m	
0	1.88±0.2	NA	NA	NA	0.92±0.0	NA	NA	NA	
8	NA	2.11±1.0	1.42±0,3	2.25±0,3	NA	0.94±0.1	1.22±0.2	0.93±0.1	
28	NA	2.16±0.3	1.62±0.1	2.26±0.3	NA	0.84±0.3	1.29±0.1	1.16±0.3	
35	NA	1.92±0.2	1.63±0.2	2.15±0.3	NA	1.09±0.3	1.13±0.2	1.22±0.2	
45	NA	1.73±0.2	1.85±0.2	2.62±0.5	NA	1.03±0.5	1.18±0.2	1.11±0.1	
	**1.5% G-MTF**				**2.0% G-MTF**				**0% G-MTF**
°C	Just prepared	1 m	3 m	6 m	Just prepared	1 m	3 m	6 m	
0	0.82±0.1	NA	NA	NA	0.73±0.2	NA	NA	NA	12.4±1.4
8	NA	0.85±0.2	0.81±0.3	0.92±0.2	NA	0.67±0.1	0.72±0.31	ND	
28	NA	0.93±0.2	0.94±0.2	1.02±0.2	NA	0.74±0.2	0.63±0.32	ND	
35	NA	1.15±0.1	0.95±0.2	1.10±0.3	NA	0.75±0.2	0.77±0.41	ND	
45	NA	1.15±0.2	1.10±0.1	1.21±0.2	NA	0.81±0.2	0.92±0.42	ND	

Table S11. Selectivity index (SI), calculated as CC_{50}/IC_{50} (promastigotes).

	**0.5% G-MTF**				**1.0% G-MTF**				
°C	Just prepared	1 m	3 m	6 m	Just prepared	1 m	3 m	6 m	
0	1.88	NA	NA	NA	1.21	NA	NA	NA	
8	NA	1.85	1.29	1.49	NA	1.25	1.85	1.22	
28	NA	2.12	1.27	1.35	NA	1.31	1.54	1.66	
35	NA	1.71	0.85	0.90	NA	1.43	1.82	0.74	
45	NA	1.78	1.02	1.39	NA	1.37	1.90	0.64	
	**1.5% G-MTF**				**2.0% G-MTF**				**0% G-MTF**
°C	Just prepared	1 m	3 m	6 m	Just prepared	1 m	3 m	6 m	
0	2.41	NA	NA	NA	2.61	NA	NA	NA	>1.23
8	NA	2.43	2.19	2.00	NA	2.58	2.67	ND	
28	NA	2.66	2.14	2.55	NA	2.24	2.17	ND	
35	NA	3.03	2.26	1.80	NA	2.34	2.08	ND	
45	NA	2.67	1.69	1.03	NA	3.00	1.64	ND	

Figure S1. Scanning electron microscopy images of 0.5% MTF carbopol-based gels under different storage conditions. The figure shows SEM images of freshly prepared 0.5% G-MTF carbopol-based gels (magnifications: 12,000×, 50,000×) vehicle (*12,000×), G-MTF stored at 8 °C (12,000×, 50,000×, and 12,000×), stored at 28 °C (magnifications: 3,000×, 100,000×, and 6,000×) and stored at 32 °C (magnifications: 3,000×, 16,000×, and 3,000×) for 4 months.

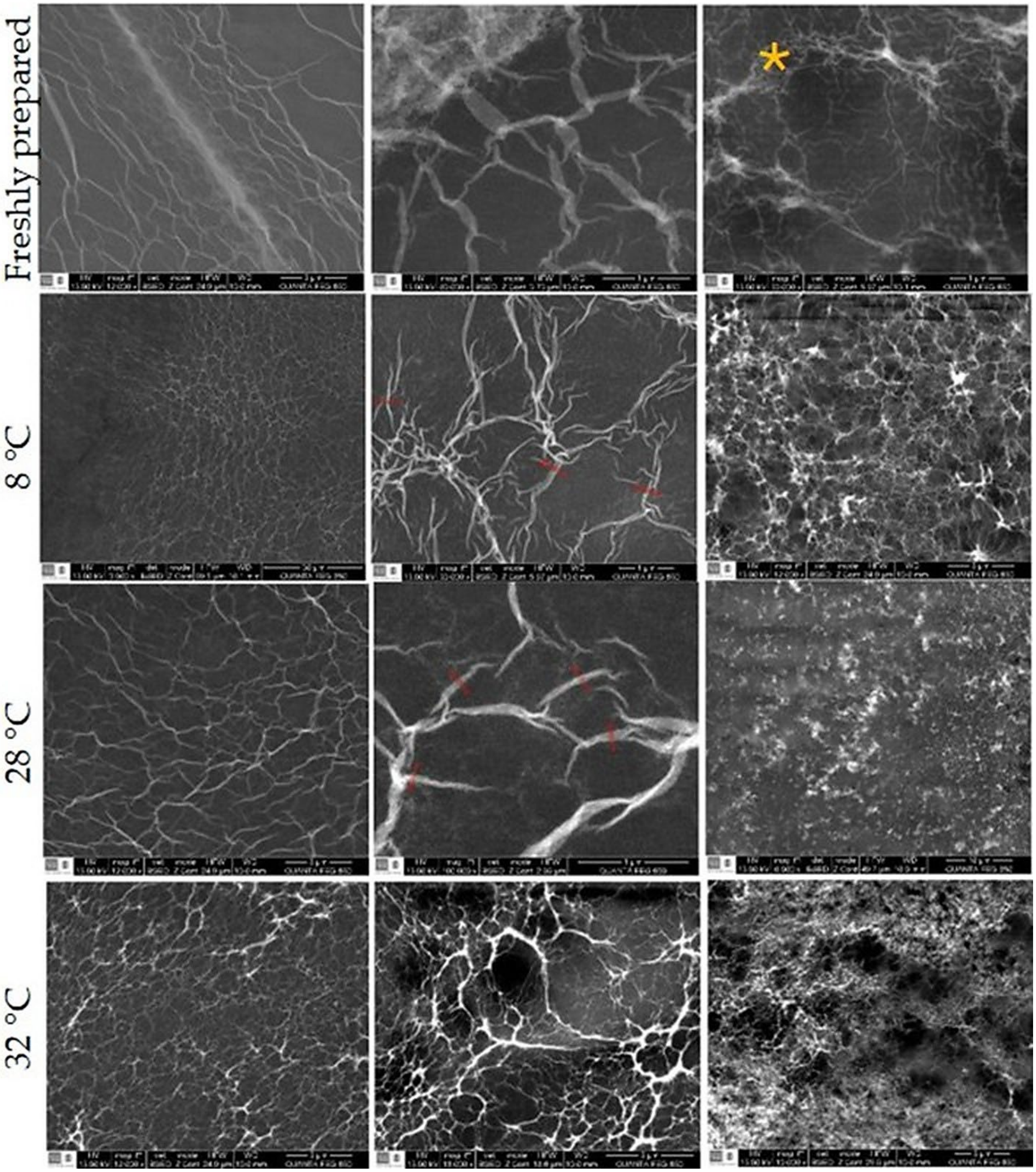

Figure S2. Influence of storage temperature (8, 28, 35, and 45 °C) and incubation time (1−6 months) on the in vitro parasite and cell activities. (a-c) Antileishmanial activity of G-MTF formulations against *L. amazonensis* promastigotes, and (d) cell toxicity activities of the gels against THP-1 cells. Lower IC_{50} values indicate greater antileishmanial activity.

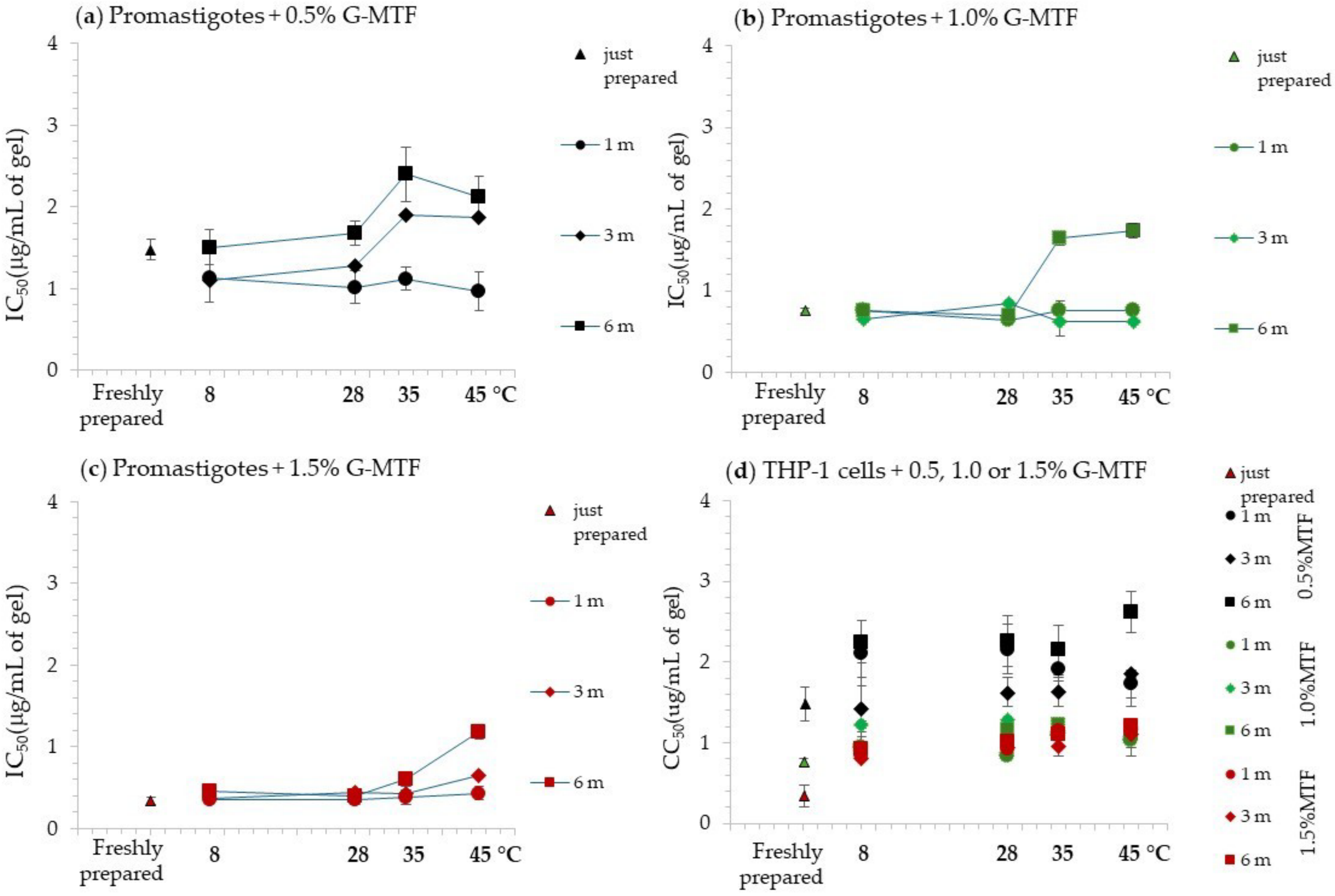

Figure S3. Time-dependent dose–response analysis of G-MTF in BALB/c mice with CL. Dose–response curves showing the relationship between the percentage of lesion reduction and the log of MTF (mM) during treatment and follow-up. Curves correspond to treatment days 8 (**a**), 16 (**b**), and 25 (**c**), and post-treatment days 8 (**d**), 16 (**e**), 24 (**f**), 32 (**g**), and 48 (h). Panel **(i**) summarises the results. Dashed red lines indicate the extrapolation used to estimate the ED_{50} and ED_{90} values, representing the miltefosine dose (mg/kg) required to achieve 50% and 90% reductions in lesion size, respectively.

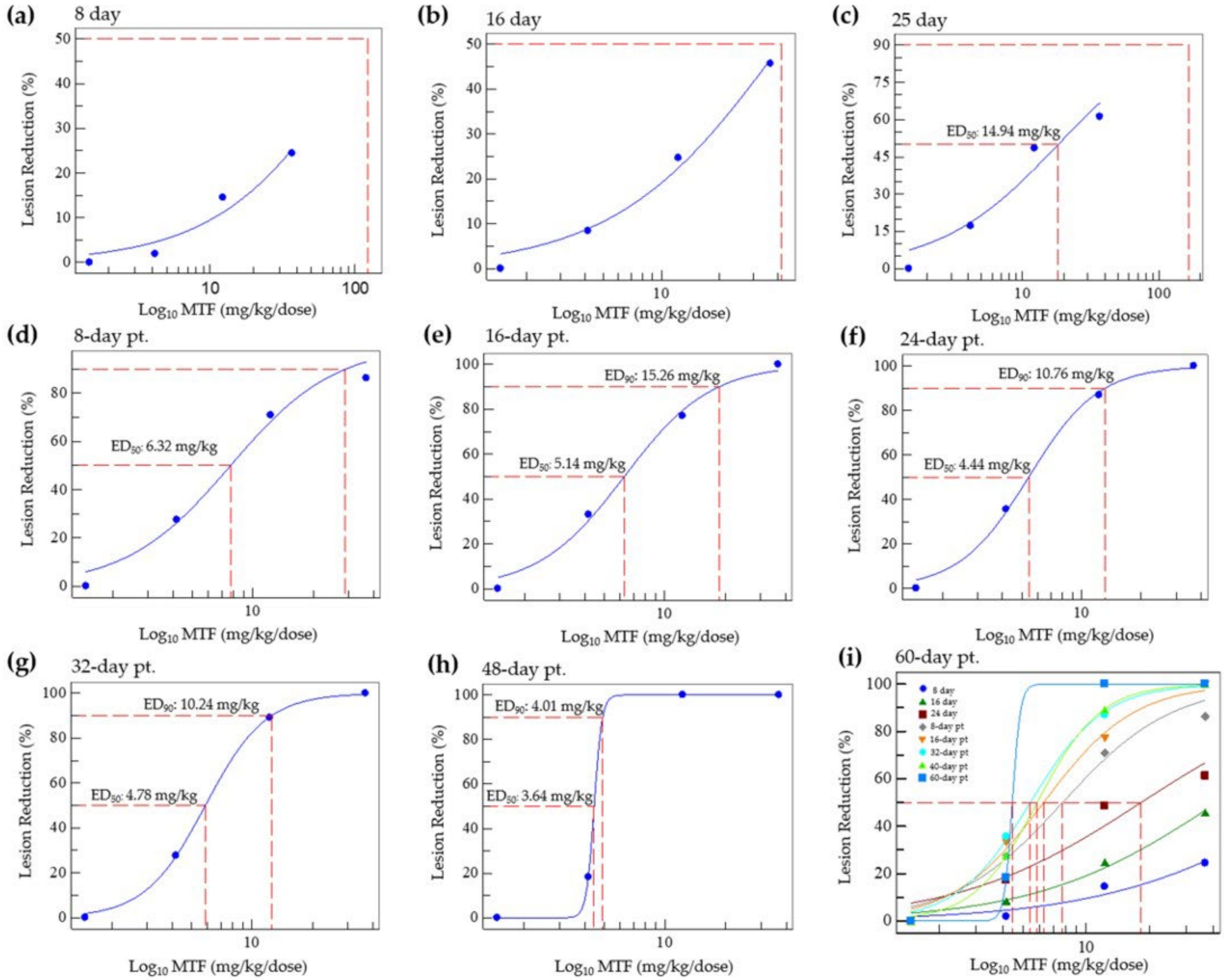

Supplement: Supplementary file 1 [file gels-12-00779-s001.zip › gels-4487820-supplementary.pdf]
